# MicroRNA-33b Inhibits Breast Cancer Metastasis by Targeting HMGA2, SALL4 and Twist1

**DOI:** 10.1038/srep09995

**Published:** 2015-04-28

**Authors:** Yancheng Lin, Allan Yi Liu, Chuannan Fan, Hong Zheng, Yuan Li, Chuankai Zhang, Shasha Wu, Donghong Yu, Zhengjie Huang, Fan Liu, Qi Luo, Chaoyong James Yang, Gaoliang Ouyang

**Affiliations:** 1 State Key Laboratory of Cellular Stress Biology, Innovation Center for Cell Signaling Network, School of Life Sciences, Xiamen University, Xiamen 361102, China; 2 Department of Surgical Oncology, First Affiliated Hospital of Xiamen University, Xiamen 361003, China; 3 Medical College, Xiamen University, Xiamen 361102, China; 4 College of Chemistry and Chemical Engineering, Xiamen University, Xiamen 361005, China

## Abstract

MicroRNAs are a class of small noncoding RNAs that regulate gene expression post-transcriptionally either by inhibiting protein translation or by causing the degradation of target mRNAs. Current evidence indicates that miR-33b is involved in the regulation of lipid metabolism, cholesterol homeostasis, glucose metabolism and several human diseases; however, whether miR-33b contributes to the pathogenesis of human cancers and participates in the regulation of self-renewal of human cancer stem cells remains unknown. Here, we report the identification of miR-33b as a negative regulator of cell stemness and metastasis in breast cancer. Compared with paired normal breast tissues, miR-33b expression is downregulated in breast tumor samples and is inversely correlated with lymph node metastatic status. Ectopic overexpression of miR-33b in highly metastatic breast cancer cells suppresses cell self-renewal, migration and invasion *in vitro* and inhibits lung metastasis *in vivo*. Conversely, miR-33b knockdown promotes the self-renewal, migration and invasion capabilities of noncancerous mammary epithelial cells. The mechanism through which miR-33b inhibits the stemness, migration and invasion of breast cancer cells is by targeting HMGA2, SALL4 and Twist1. These data indicate that miR-33b acts as an onco-suppressive microRNA in breast cancer progression by inhibiting the stemness and metastasis of breast cancer cells.

Breast cancer is one of the most prevalent cancers in women and accounts for approximately 22.9% of all cancers in women world-wide. Breast cancer is also ranked as the 2^nd^ leading cause of death in women after lung cancer. In 2013 in the United States, ~232,340 women were diagnosed with invasive breast cancer and ~39,620 died from the disease^1^. In China, breast cancer is also one of the most common cancers in women, and its incidence has increased by 3% annually^2^. Nearly half of breast cancer patients develop distant metastatic disease after treatment with chemotherapeutic and/or hormonal drugs. The mortality of breast cancers has decreased by 34% and the five-year overall survival rate has increased to 90% since 1990^3^; however, metastatic breast cancer remains difficult to cure. Despite the significant advances that have been achieved in understanding breast cancer metastasis in the past decades, the molecular mechanism underlying its metastasis remains elusive.

Cancer stem cells (CSCs) are a subpopulation of cancer cells that can self-renew and reconstitute the hierarchical organization of tumors, whereas their counterpart non-CSCs are not tumorigenic^4^,^5^. CSCs have been identified and purified from nearly all types of solid tumors, such as breast cancer^6^, colon cancer^7^, brain tumor^8^ and melanoma^9^. Accumulating evidence has suggested that CSCs contribute to various aspects of tumor pathogenesis, including tumor initiation, metastasis and recurrence, as well as tumor heterogeneity^10^,^11^. However, our understanding of the regulators that mediate the stemness of CSCs in these processes is far from complete.

MicroRNAs (miRNAs) are a class of endogenous 21- to 23-nucleotide non-coding RNAs that regulate gene expression by directly binding to mRNAs to modulate mRNA degradation or inhibit translation^12^,^13^. The differential expression of miRNAs has been reported in different types of cancers^14^. Moreover, specific miRNAs can act as promoters or inhibitors of the progression of metastasis and coordinately suppress numerous target genes in the invasion-metastasis cascade. Recent studies have discovered that miRNAs are involved in multiple steps and phenotypes of breast cancer progression, such as the epithelial-mesenchymal transition (EMT)^15^,^16^, stemness^17^, invasion and metastasis^18^,^19^. In this report, we show that miR-33b is downregulated in highly metastatic breast cancer cell lines as well as breast cancer tissue samples. The miR-33b expression levels are negatively correlated with clinical stage and lymph node metastasis of human breast cancer. Using *in silico* and other analyses, we demonstrate that HMGA2, SALL4 and Twist1 are *bona fide* downstream targets of miR-33b. Moreover, we report that miR-33b can regulate the stem cell properties of breast cancer cells. We also reveal that miR-33b inhibits cell migration and invasion *in vitro* and lung metastasis *in vivo*. Therefore, our findings provide evidence for miR-33b as a novel diagnostic and prognostic marker for breast cancer metastasis.

## Results

### miR-33b is downregulated in breast cancer

**Figure 1:**
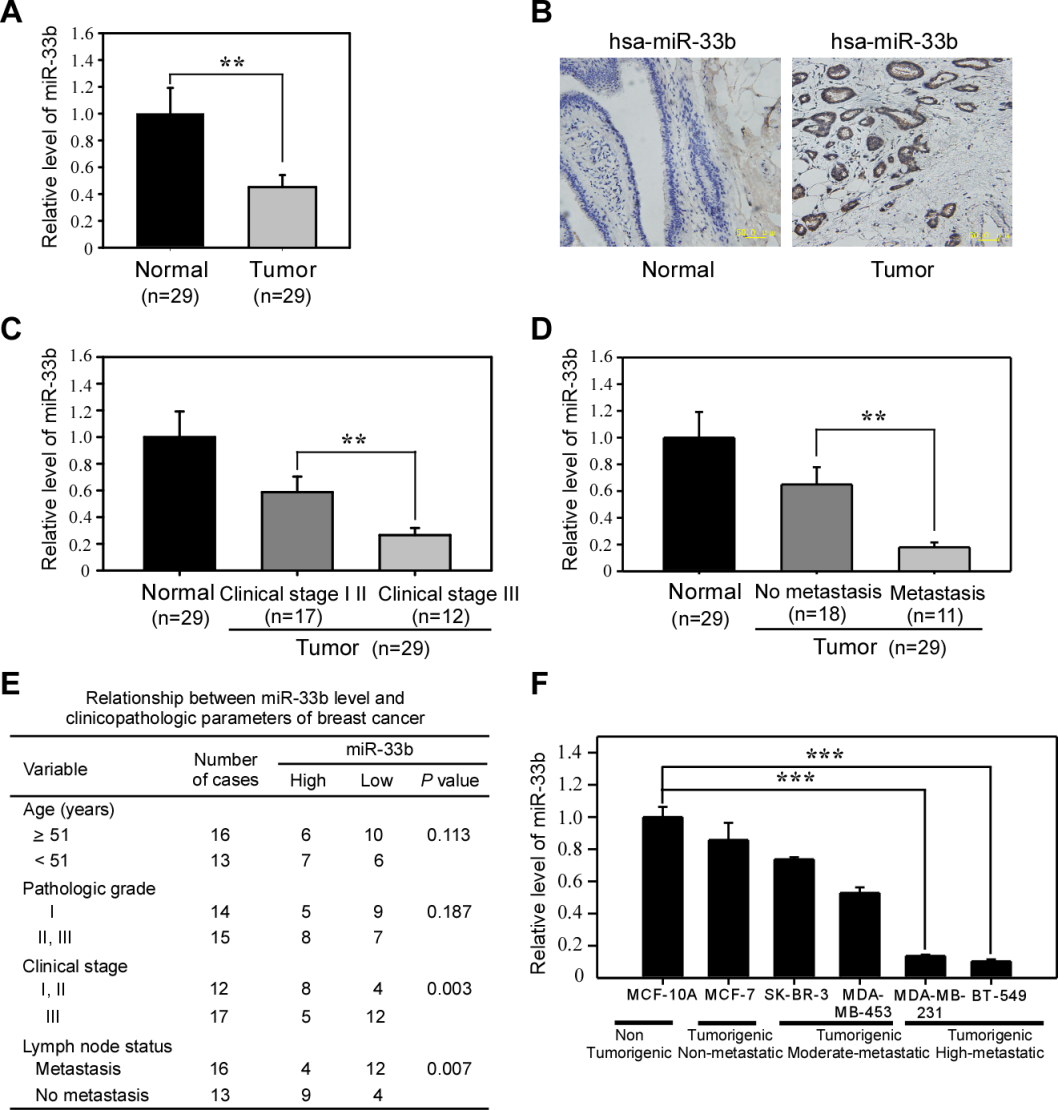
miR-33b is downregulated in breast cancer tissue samples and breast cancer cell lines. (A) qRT-PCR analysis of miR-33b expression in human breast cancer tissue samples and their matched normal breast tissues from 29 breast cancer patients. (B) *In situ* hybridization analysis of miR-33b expression in human breast cancer tissues and matched normal tissues. (C) Correlation between miR-33b expression and the progression of the clinical stage of breast cancer. (D) Correlation between miR-30b expression and the lymph node metastasis status of breast cancer. (E) Correlation between clinicopathological features and miR-33b expression in 29 breast cancer tissues. (F) qRT-PCR analysis of miR-33b expression in noncancerous human mammary epithelial cells and breast cancer cell lines with different metastatic potential. Scale bars, 50 μm. Data represent the mean ± s.d. **: *P* <0.01, ***: *P* <0.001.

miR-33b is a member of the human miR-33 family and is located in intron 17 of the human SREBP-1 gene^20^,^21^. miR-33b has been reported to regulate lipid metabolism and cholesterol homeostasis by targeting the downstream genes ABCA1, AMPK and SIRT6^22^. However, whether miR-33b plays functional roles in tumorigenesis and metastasis remains unclear. To determine the role of miR-33b in breast cancer progression, we examined miR-33b expression in breast cancer tissue samples by qRT-PCR. miR-33b was downregulated in the breast cancer samples compared with the paired normal breast tissues (Fig. 1A). *In situ* hybridization analysis also revealed that miR-33b expression in human breast cancer tissues was much lower than in matched normal tissues (Fig. 1B).

Moreover, the levels of miR-33b were negatively correlated with the progression of clinical stage (Fig. 1C) and lymph node metastasis status (Fig. 1D). The correlation between the miR-33b expression level and clinical and pathologic characteristics of breast cancer is summarized in Fig. 1E. In 17 cases presenting as advanced stage III, 12 (70.59%) of the cases have low-level miR-33b expression in cancer tissues; however, in 12 early stages (stages I and II), only 4 (33.33%) presented with low levels of miR-33b expression. In the 16 cases of breast cancer patients with lymph node metastasis, 12 (75%) exhibited low miR-33b expression, while only 4 (30.77%) of 13 cases of cancers without lymph node metastasis presented low-level miR-33b expression. No correlation was observed between the miR-33b level and the age or pathologic grade status of breast cancer. We further investigated miR-33b expression in the noncancerous human mammary epithelial cell line MCF-10A and in the following breast cancer cell lines: the non-metastatic cell line MCF-7, moderately metastatic cell lines SK-BR-3 and MDA-MB-453, and highly metastatic cell lines BT-549 and MDA-MB-231. Compared with the noncancerous breast epithelial cell line MCF-10A, miR-33b expression was significantly downregulated in the highly metastatic breast cancer cell lines MDA-MB-231 and BT-549 (Fig. 1F). Altogether, these data demonstrate that miR-33b is downregulated in breast cancer and that its expression is inversely correlated with the metastatic abilities of breast cancer cells.

### HMGA2, SALL4 and Twist1 are *bona fide* downstream targets of miR-33b in breast cancer cells

**Figure 2:**
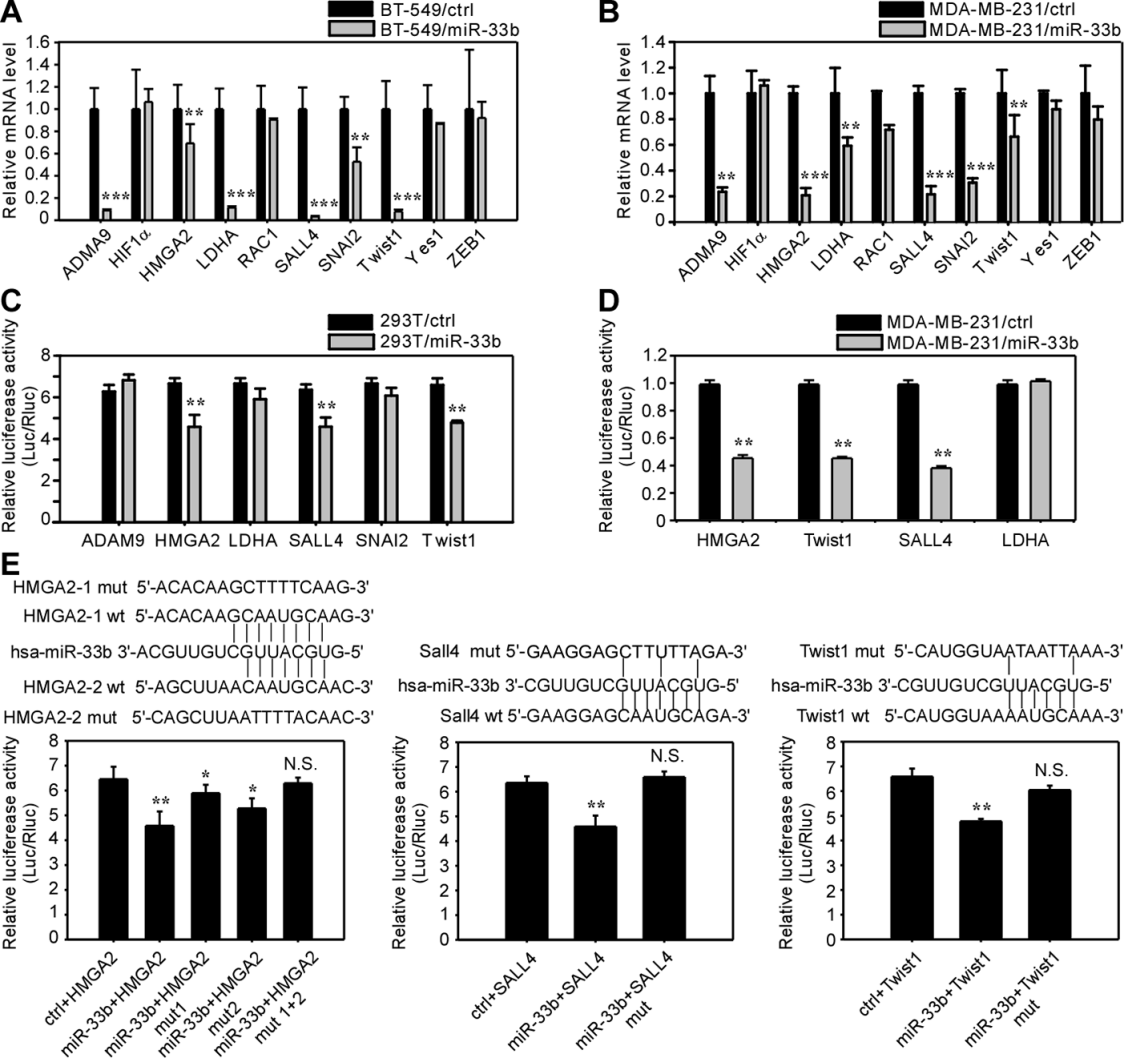
HMGA2, SALL4 and Twist1 are *bona fide* downstream targets of miR-33b. (A) qRT-PCR analysis of the mRNA levels of predicted target genes in BT-549/ctrl and BT-549/miR-33b cells. (B) qRT-PCR analysis of the mRNA levels of predicted target genes in MDA-MB-231/ctrl and MDA-MB-231/miR-33b cells. (C) Dual luciferase reporter assay analysis of the effect of miR-33b expression on the activity of the 3′UTR of target genes in 293T cells. (D) Dual luciferase reporter assay analysis of the effect of miR-33b expression on the activity of the 3′UTR of target genes in MDA-MB-231 cells. (E) Dual luciferase reporter assay analysis of the effect of miR-33b expression on the activity of wild-type and mutant 3′UTRs of HMGA2, SALL4 and Twist1. Data represent the mean ± s.d. *: *P* <0.05, **: *P* <0.01, ***: *P* <0.001, N.S.: no significance.

To decipher the regulatory role of miR-33b in breast cancer, we aimed to identify direct downstream targets of miR-33b and to further investigate its underlying molecular mechanism as a tumor-suppressive miRNA. To narrow down the target genes of miR-33b, we employed different analytic strategies. First, we used three *in silico* algorithms (Targetscan, miRanda and Pictar) to predict miR-33b target genes with high binding possibilities^23^. Second, we used qRT-PCR to screen putative miR-33b targets with more than 30% of reduced expression upon miR-33b overexpression in MDA-MB-231 and BT-549 cells. Finally, we cloned the wild-type and mutant 3′UTRs of these candidate target genes into luciferase constructs to examine whether miR-33b can directly bind to these mRNAs. After the initial screening of target genes using online databases and two confirmed miR-33b target genes ABCA1 and SIRT6 as a reference for screening, we obtained the following candidates: ADAM9, HIF-1α, HMGA2, LDHA, RAC1, SALL4, SNAI2, Twist1, Yes1 and ZEB1. Most of these candidates are oncogenes that regulate EMT, metastasis or stemness in various cancers. We performed qRT-PCR to analyze the endogenous mRNA levels of these genes upon the overexpression of miR-33b in BT-549 and MDA-MB-231 cells (Supplementary Fig. 1). The ectopic expression of miR-33b downregulated the expression of ADAM9, HMGA2, LDHA, SALL4, SNAI2 and Twist1 by more than 30% but had minimal effects on HIF-1α, RAC1, Yes1 and ZEB1 in these two breast cancer cell lines (Fig. 2A,B). Next, we cloned each 3′UTR of these 6 genes into pmiR-Report constructs and performed dual luciferase reporter assays to investigate whether miR-33b could directly regulate the expression of these genes. As shown in Fig. 2C,D, the overexpression of miR-33b dramatically decreased the luciferase activity of HMGA2, SALL4 and Twist1 by 25-50% but did not alter the luciferase activity of ADAM9, LDHA and SNAI2.

To further determine whether miR-33b could regulate the expression of these genes by directly binding to miRNA-responsive elements in the 3′UTR, we mapped the miR-33b binding sites in the 3′UTR of HMGA2, SALL4 and Twist1. We found two putative binding sites in the HMGA2 3′UTR, one putative binding site in the SALL4 3′UTR and one putative miR-33b binding site in the Twist1 3′UTR and then used Quickchange PCR^24^ to obliterate these binding sites in the 3′UTR of HMGA2, SALL4 and Twist1. We found that the mutation of the 3′UTR binding sites of miR-33b target genes SALL4 and Twist1 abrogated the suppressive effect on the luciferase activity induced by the ectopic expression of miR-33b (Fig. 2E). For HMGA2, which has two potential miR-33b binding sites, mutation of either binding site 1 or binding site 2 reversed the decreased luciferase activity induced by miR-33b. However, the first binding site displayed a stronger suppressive effect than the second binding site. All of these data suggest that Twist1, HMGA2 and SALL4 are *bona fide* downstream targets of miR-33b.

### miR-33b inhibits the stem cell-like properties of breast cancer cells *in vitro*

**Figure 3:**
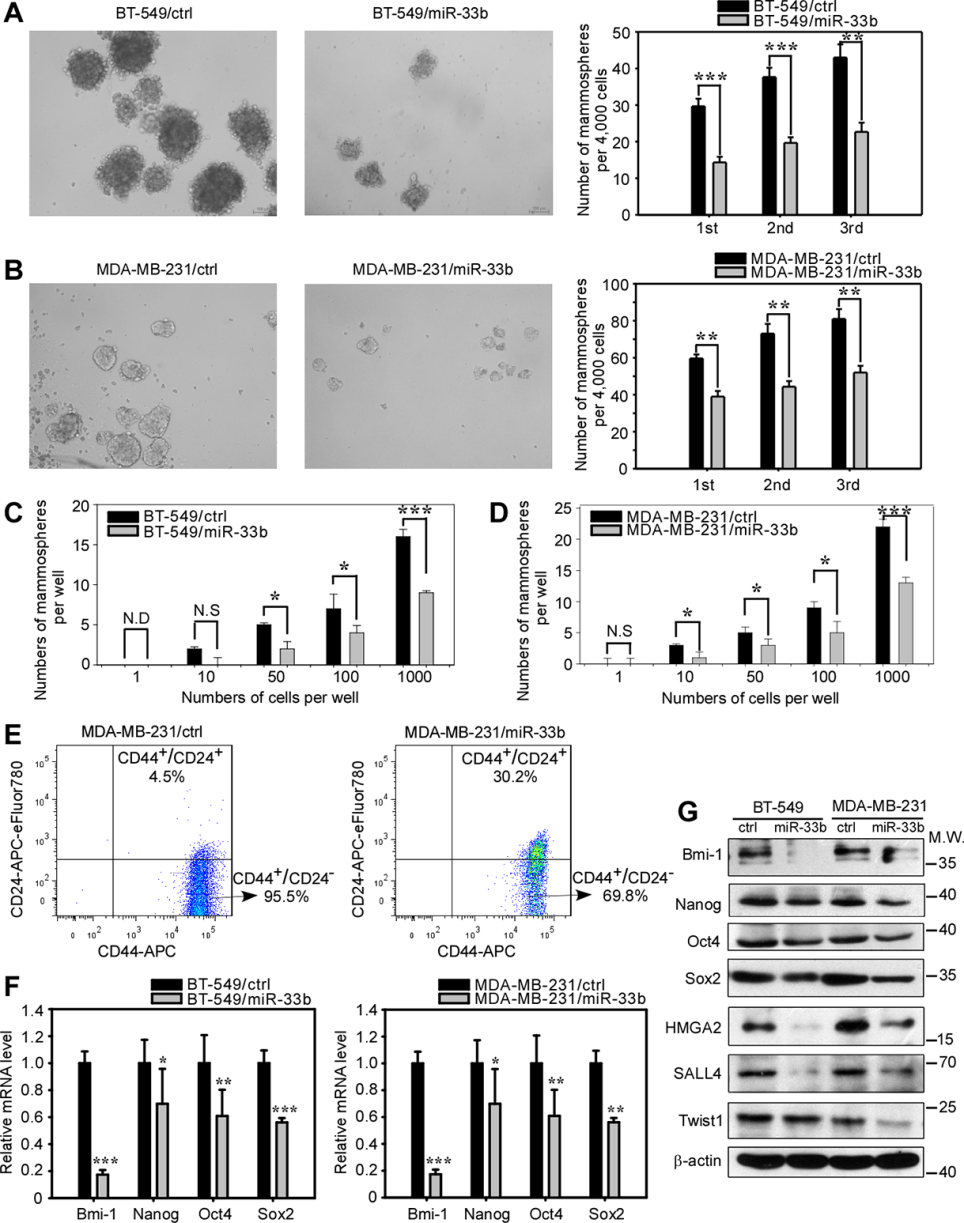
miR-33b inhibits breast cancer cell stemness. (A) Representative images of the mammospheres formed by BT-549/ctrl and BT-549/miR-33b cells. Quantification of primary, secondary and tertiary mammospheres formed by BT-549/ctrl and BT-549/miR-33b cells. (B) Representative images of the mammospheres formed by MDA-MB-231/ctrl and MDA-MB-231/miR-33b cells. Quantification of primary, secondary and tertiary mammosphere formation formed by MDA-MB-231/ctrl and MDA-MB-231/miR-33b cells. (C,D) Tumorsphere assays for BT-549/ctrl and BT-549/miR-33b (C) and MDA-MB-231/ctrl and MDA-MB-231/miR-33b (D) cells were performed by limiting dilution with 1,000 cells to one cell per well of a 96-well ultra-low attachment plate. The numbers of mammospheres were scored at the end of 7 days. This experiment was performed three times, with four wells per cell dilution. (E) FACS analysis of the CD44^+^/CD24^−^ stem cell subpopulation in MDA-MB-231/ctrl and MDA-MB-231/miR-33b cells. (F) qRT-PCR analysis of the mRNA expression of stemness-related genes Bmi-1, Nanog, Oct4 and Sox2 in BT-549/ctrl and BT-549/miR-33b cells as well as in MDA-MB-231/ctrl and MDA-MB-231/miR-33b cells. (G) Western blot analysis of Bmi-1, Nanog, Oct4, Sox2, HMGA2, SALL4 and Twist1 expression in BT-549/ctrl and BT-549/miR-33b cells as well as in MDA-MB-231/ctrl and MDA-MB-231/ miR-33b cells. The full-length blots were presented in the Supplementary Figure 8. Scale bars, 100 μm. Data represent the mean ± s.d. *: *P* <0.05, **: *P* <0.01, ***: *P* <0.001, N.S.: no significance, N.D.: not detected.

After having identified these three main downstream targets of miR-33b, we further examined which specific phenotypes are regulated by miR-33b in breast cancer pathogenesis. Previous studies have revealed that HMGA2, SALL4 and Twist1 are involved in cancer stem cell self-renewal in different cell types. Therefore, we first addressed whether miR-33b could suppress the stemness of breast cancer cells. We employed a well-defined mammosphere formation assay in non-adherent non-serum medium to examine cell stemness^25^. The ectopic expression of miR-33b in BT-549 and MDA-MB-231 cells decreased the size and number of mammospheres in primary, secondary and tertiary cultures (Fig. 3A,B). We further employed a limiting dilution assay to determine the stem cell frequency. At the 1-cell level, ectopic expression of miR-33b in BT-549 and MDA-MB-231 cells showed no difference in sphere formation compared with their control cells. However, at the 10-, 50-, 100- and 1,000-cell levels, BT-549/ctrl and MDA-MB-231/ctrl cells formed more mammospheres than BT-549/miR-33b and MDA-MB-231/miR-33b cells (Fig. 3C,D), indicating that miR-33b inhibits the self-renewal of breast CSCs *in vitro*.

The CD44^+^/CD24^−^ immunophenotype has been successfully used as a biomarker to identify cancer stem-like cells in breast cancer cell populations^6^. Therefore, we used fluorescence-activated cell sorting (FACS) to analyze whether ectopic miR-33b expression modulated the CSC subpopulation in MDA-MB-231 cells. However, we did not use FACS to analyze the CD44^+^/CD24^−^ subpopulation in BT-549 cells because its CD44^+^/CD24^−^ subpopulation is very low (2.3%). As shown in Fig. 3E, the ectopic expression of miR-33b abated the CD44^+^/CD24^−^ subpopulation of MDA-MB-231 cells from 95.5% to 69.8%. These FACS results suggested that miR-33b can decrease the cancer stem-like cell pool of breast cancer cells. Next, we investigated the impact of miR-33b on the expression of stem markers. We used qRT-PCR to analyze the mRNA level of the stem cell markers Oct4, Sox2, Bmi-1 and Nanog in BT-549 and MDA-MB-231 cells and found that miR-33b expression decreased the expression of these genes at the mRNA level (Fig. 3F). We further employed western blot analysis to examine the miR-33b downstream targets and expression of these stemness markers at the protein level. The overexpression of miR-33b suppressed the expression of the miR-33b targets HMGA2, SALL4 and Twist1, as well as the stemness-related proteins Bmi-1, Nanog, Oct4 and Sox2 (Fig. 3G), in agreement with the qRT-PCR results. Collectively, these data indicate that miR-33b can inhibit breast cancer stem-like cell self-renewal by targeting HMGA2, SALL4 and Twist1.

### miR-33b suppresses breast cancer cell migration and invasion *in vitro*

**Figure 4:**
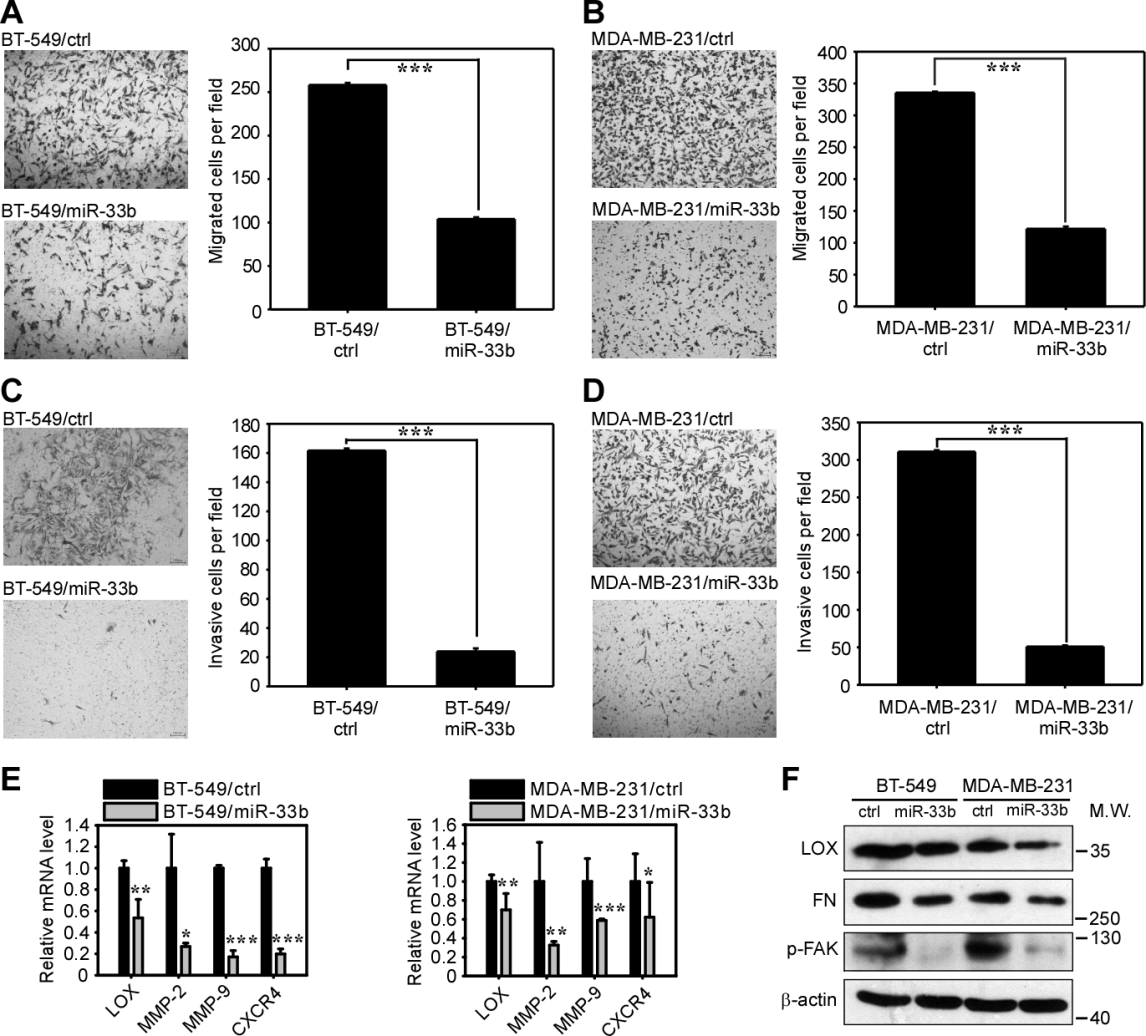
miR-33b suppresses the migration and invasion of breast cancer cells *in vitro*. (A,B) Transwell migration assays revealed that the ectopic expression of miR-33b inhibited the migration ability of BT-549 (A) and MDA-MB-231 (B) cells. (C,D) Matrigel-coated Transwell invasion assay revealed that the ectopic expression of miR-33b inhibited the invasion ability of BT-549 (C) and MDA-MB-231 (D) cells. (E) qRT-PCR analysis revealed that miR-33b downregulated the mRNA expression of metastasis-related genes LOX, MMP-2, MMP-9 and CXCR4 in BT-549 and MDA-MB-231 cells. (F) Western blot analysis of the metastasis-related proteins LOX, FN and p-FAK. miR-33b downregulated the levels of these proteins in BT-549 and MDA-MB-231 cells. The full-length blots were presented in the Supplementary Figure 9. Scale bars, 100 μm. Data represent mean ± s.d. *: *P* <0.05, **: *P* <0.01, ***: *P* <0.001.

Recently, CSCs have been linked to tumor metastasis capacity. In breast cancer, the CD44^+^/CD24^−^ subpopulation, which refers to breast cancer stem-like cells, showed enriched metastatic abilities in a xenograft model^6^. Moreover, Mani *et al*. discovered that CSCs expressed some EMT markers and had the ability to migrate^26^, leading to the concept that CSCs may contribute to the pool of metastasis- initiating cells^27^. Because miR-33b inhibited breast cancer stem-like cell self-renewal, and its expression is inversely related to the metastatic potential in breast cancer cell lines, we further addressed whether miR-33b could suppress the migration and invasive abilities of breast cancer cells. We used Transwell assays to evaluate the migration and invasion capacities *in vitro*. The ectopic expression of miR-33b in BT-549 and MDA-MB-231 cells dramatically suppressed cell migration (Fig. 4A,B) and invasion (Fig. 4C,D). These defects could not be ascribed to toxicity resulting from ectopic miR-33b (Supplementary Fig. 2A,B). These data suggest that miR-33b can suppress the migration and invasion of breast cancer cells *in vitro*.

We further asked how miR-33b regulated breast cancer cell migration and invasion. Initially, we focused on the three major downstream targets of miR-33b: HMGA2, SALL4 and Twist1. Twist1 is a well-defined pro-metastatic transcription factor that can induce EMT to promote invasion and metastasis^26^. A previous study also revealed that Twist1 could enhance cell movement beyond EMT, with Twist1 inducing the activation of Rac1^28^. Twist1 has been reported to regulate MMP-2 and MMP-9 expression to regulate matrix remodeling in hepatocellular carcinoma^29^. HMGA2 also plays important roles in regulating invasion and metastasis and has been shown to downregulate miR-200b to increase the level of lysyl oxidase (LOX) to promote invasiveness in breast cancer^30^. HMGA2 can enhance breast cancer metastasis by upregulating the pro-metastatic gene CXCR4^31^. Thus, we examined whether the ectopic expression of miR-33b could induce the mesenchymal-epithelial transition (MET) in BT-549 and MDA-MB-231 cells. We employed western blotting to detect the protein expression of the epithelial cell markers α-E-catenin, E-cadherin and β-catenin and mesenchymal cell markers vimentin, N-cadherin and α-SMA. Upon the overexpression of miR-33b, we did not detect any significant changes in these proteins, indicating that miR-33b did not inhibit migration and invasion through MET (Supplementary Fig. 3A). Because both Twist1 and HMGA2 can regulate the proteins responsible for ECM degradation, we speculated that miR-33b may regulate migration and invasion in breast cancer cells via matrix remodeling. Next, we used qRT-PCR to analyze the mRNA level of metastasis-related genes that have been reported to be regulated by Twist1 and HMGA2. We found that the ectopic expression of miR-33b in BT-549 and MDA-MB-231 cells decreased the expression of MMP-2, MMP-9, LOX and CXCR4 (Fig. 4E). We further employed western blotting to detect the protein expression of downstream metastatic genes of miR-33b. Because HMGA2 regulates LOX expression, we continued to detect the downstream effectors of LOX in breast cancers. LOX can regulate p-FAK to promote cell migration and invasion^32^ and support cell adhesion to fibronectin (FN); thus, we used western blotting to examine the protein levels of LOX, FN and p-FAK, which were dramatically downregulated upon miR-33b expression (Fig. 4F). Altogether, these data showed that miR-33b suppresses cell migration and invasion *in vitro* by regulating Twist1 and HMGA2.

### Knockdown of miR-33b promotes the self-renewal, migration and invasion of MCF-10A cells

**Figure 5:**
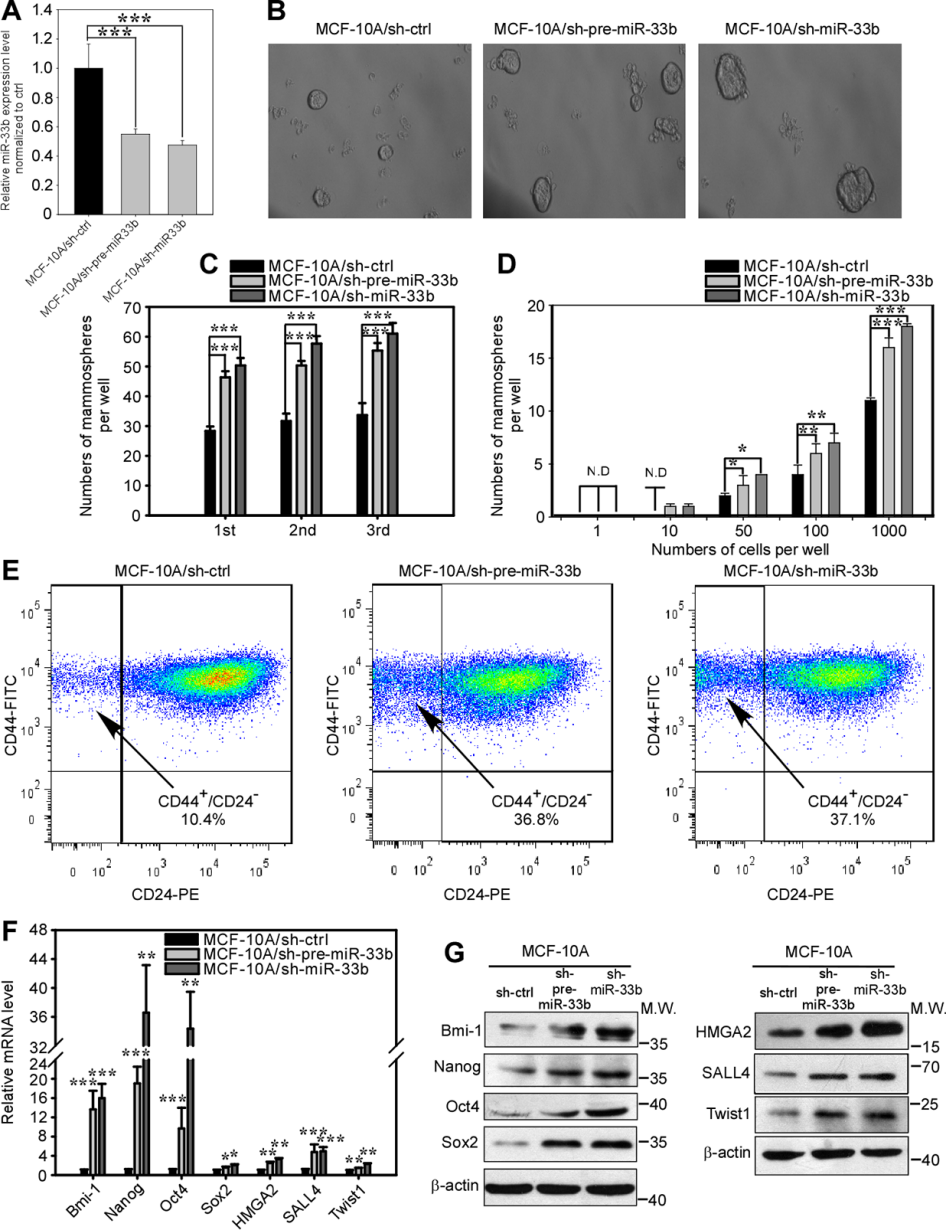
Knockdown miR-33b promotes the self-renewal of MCF-10A cells. (A) qRT-PCR analysis of the knockdown efficiency of miR-33b in MCF-10A cells. (B) Representative images of the mammospheres formed by MCF-10A/sh-ctrl, MCF-10A/sh-pre-miR-33b and MCF-10A/sh-miR-33b cells. (C) Quantification of primary, secondary and tertiary mammospheres formed by MCF-10A/sh-ctrl, MCF-10A/sh-pre-miR-33b and MCF-10A/sh-miR-33b cells. (D) A limiting dilution assay was performed on the MCF-10A/sh-ctrl, MCF-10A/sh-pre-miR-33b and MCF-10A/sh-miR-33b from 1 to 1,000 cells per well. The numbers of mammospheres were scored at the end of 7 days. (E) FACS analysis of the CD44^+^/CD24^−^ stem cell subpopulation in MCF-10A/sh-ctrl, MCF-10A/sh-pre-miR-33b and MCF-10A/sh-miR-33b cells. (F) qRT-PCR analysis of the mRNA expression of stemness-related genes Bmi-1, Nanog, Oct4 and Sox2 as well as the downstream target genes of miR-33b, HMGA2, SALL4 and Twist1 in MCF-10A/sh-ctrl, MCF-10A/sh-pre-miR-33b and MCF-10A/sh-miR-33b cells. (G) Western blot analysis of the protein expression of stemness-related genes Bmi-1, Nanog, Oct4 and Sox2 as well as the downstream target genes of miR-33b, HMGA2, SALL4 and Twist1. The full-length blots were presented in the Supplementary Figure 10. Scale bars, 100 μm. Data represent the mean ± s.d. *: *P* <0.05, **: *P* <0.01, ***: *P* <0.001, N.D.: not detected.

Because the overexpression of miR-33b inhibited the self-renewal, cell migration and invasion of breast cancer cells *in vitro*, we asked whether the inhibition of miR-33b enhances self-renewal and cell mobility. First, we used a lentivirus-based antagomir expression system to knockdown endogenous miR-33b in MCF-10A cells^33^. We designed two pairs of oligonucleotides specifically targeting precursor miR-33b (pre-miR-33b) and mature miR-33b. To examine the inhibitory effects of lentivirus-based antagomir expression, we performed qRT-PCR to detect the levels of miR-33b. miR-33b was significantly downregulated by approximately 50% after the lentiviral infection of anti-miR-33b and anti-pre-miR-33b (Fig. 5A). Next, we explored whether miR-33b knockdown could augment self-renewal in MCF-10A cells. Notably, the knockdown of miR-33b and pre-miR-33b dramatically increased the sphere formation ability of MCF-10A cells in a serial passage mammosphere formation assay and limiting dilution assay (Fig. 5B–D). Similar data were obtained by FACS analysis. The knockdown of miR-33b in MCF-10A cells increased the CD44^+^/CD24^−^ stem cell subpopulation from 10.4% to 36.8% for MCF-10A/sh-miR-33b cells and from 10.4% to 37.1% for MCF-10A/sh-pre-miR-33b cells (Fig. 5E). qRT-PCR and western blot analyses also revealed that the inhibition of miR-33b in MCF-10A cells upregulated the expression of the miR-33b downstream targets HMGA2, SALL4 and Twist1, as well as the stem cell markers Bmi-1, Nanog, Oct4 and Sox2 (Fig. 5F,G). Collectively, these results indicate that the inhibition of miR-33b can promote the self-renewal of MCF-10A cells.

**Figure 6:**
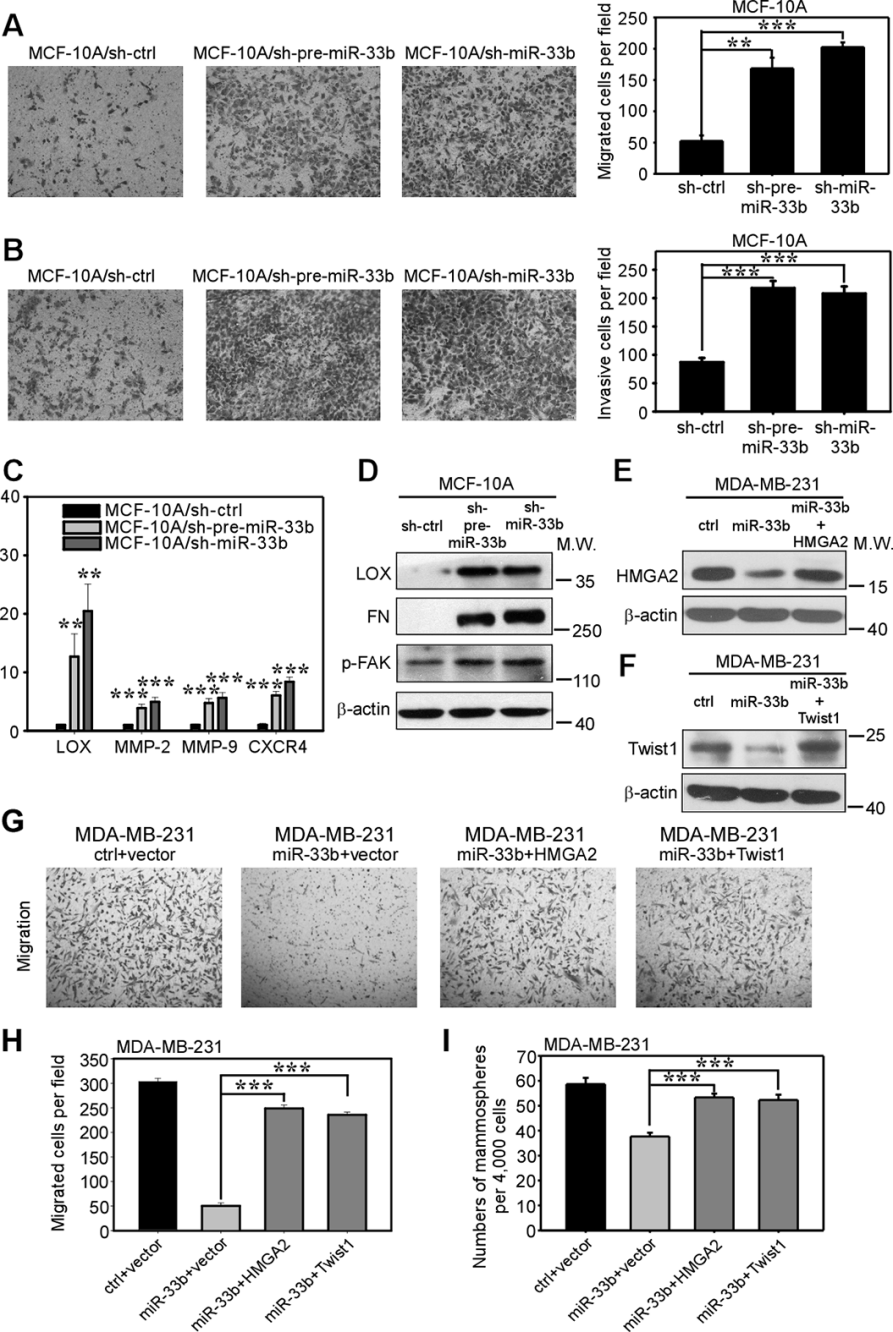
Knockdown of miR-33b promotes the migration and invasion capabilities of MCF-10A cells, and re-expression of HMGA2 and Twist1 reverses miR-33b-dependent self-renewal and invasion-relevant phenotypes of MDA-MB-231 cells. (A,B) Transwell migration (A) and Matrigel-coated Transwell invasion (B) analyses revealed that knockdown of miR-33b promoted the migration and invasion of MCF-10A cells *in vitro*. (C) qRT-PCR analysis revealed that the knockdown of miR-33b upregulated the mRNA expression of the metastasis-related genes LOX, MMP-2, MMP-9 and CXCR4 in MCF-10A cells. (D) Western blot analysis of metastasis-related proteins LOX, FN and p-FAK. These proteins were upregulated after miR-33b knockdown in MCF-10A cells. (E, F) Western blot analysis of the re-expression of HMGA2 and Twist1 in MDA-MB-231/miR-33b cells. The full-length blots were presented in the Supplementary Figure 11. (G) Migration assays with the indicated MDA-MB-231 cells transfected with miRNA-resistant expression constructs. Control represents the scrambled miRNA used for miR-33b overexpression, and vector represents the empty vector used for HMGA2 and Twist1 re-expression. (H) Quantification of migration assays with the indicated MDA-MB-231 cells transfected with miRNA-resistant expression constructs in (G). (I) Quantification of mammosphere formation assays with the indicated MDA-MB-231 cells transfected with miRNA-resistant expression constructs. Scale bars, 100 μm. Data represent mean ± s.d. **: *P* <0.01, ***: *P* <0.001.

We further investigated whether the inhibition of miR-33b in MCF-10A cells could result in a pro-invasive phenotype. EMT is a critical program for cancer cells to achieve invasive capability. However, miR-33b did not induce EMT in MCF-10A cells (Supplementary Fig. 3B). The Transwell assay showed that the inhibition of miR-33b in MCF-10A cells drastically enhanced migration (Fig. 6A) and invasion capabilities (Fig. 6B). qRT-PCR and western blotting results were consistent with the Transwell assay. The mRNA levels of the metastasis-related genes LOX, MMP-2, MMP-9 and CXCR4 were upregulated upon miR-33b inhibition in MCF-10A cells (Fig. 6C), and miR-33b knockdown also increased the protein levels of LOX, FN and p-FAK (Fig. 6D). However, knockdown of miR-33b did not exhibit a toxicity effect on MCF-10A cell viability (Supplementary Fig. 4). Altogether, these data showed that knockdown of miR-33b promotes cell migration and invasion *in vitro*.

### Re-expression of HMGA2 and Twist1 reverses miR-33b-dependent self-renewal and invasion-relevant phenotypes *in vitro*

To determine whether the phenotypes associated with ectopic miR-33b expression could be reversed via the restoration of its target gene expression, we transfected miR-33b-expressing MDA-MB-231 cells with individual expression constructs of HMGA2 or Twist1 rendered miRNA insensitive by deletion of their 3′UTRs. In miR-33b-expressing cells, both HMGA2 and Twist1 reversed, at least partially, miR-33b-imposed migration and mammosphere formation defects (Fig. 6E–I). We also analyzed cDNA microarray data in Oncomine^34^ to further confirm the role of HMGA2, SALL4 and Twist1 in breast cancer pathogenesis. As shown in Supplementary Fig. 5A–C, all of these three miR-33b target mRNAs were upregulated in invasive breast cancer tissues compared with normal breast tissues. Therefore, HMGA2, SALL4 and Twist1 are functionally relevant effectors of miR-33b.

### miR-33b acts as a metastatic suppressor *in vivo*

**Figure 7:**
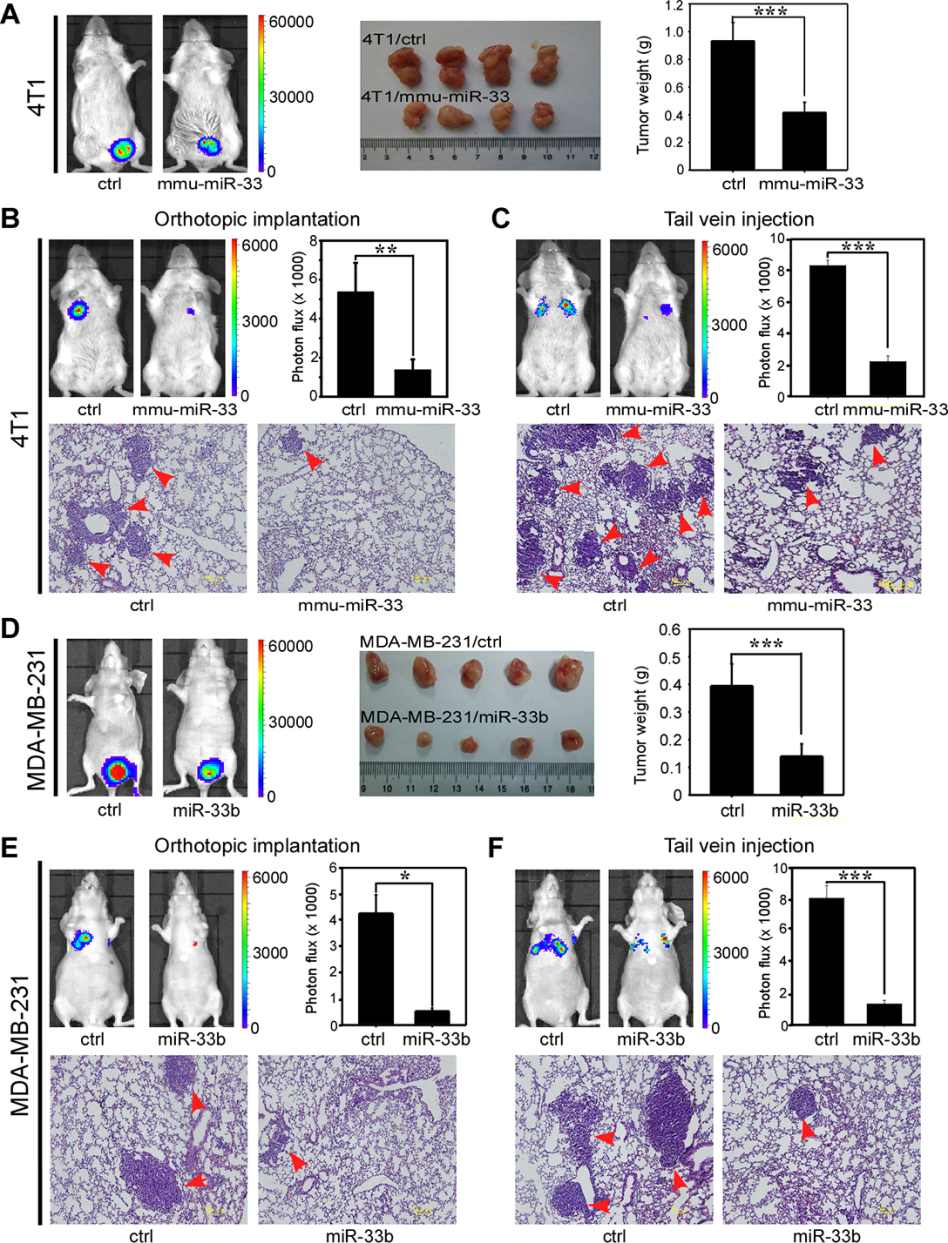
miR-33b inhibits tumor growth and lung metastasis of breast cancer cells *in vivo*. (A) Bioluminescence images of mice showing primary tumors derived from orthotopic injections into the mammary fat pad of 4T1 control cells (ctrl) or 4T1/mmu-miR-33 cells at day 21 (left). Images of tumors from all mice in each group (middle). Quantitation of tumors from mice in each group (right) (n = 4). (B) Bioluminescence images of mice showing lung metastases at day 35 derived from orthotopic injections of 4T1 control cells and mmu-miR-33-expressing cells. In this case, primary tumors were resected at day 21 (top-left). Quantitation of lung metastases as assessed by Bioluminescence measurements (top-right) (n = 4). Arrows indicate metastatic foci (bottom). (C) Bioluminescence images of animals showing lung metastases at day 14 derived from the tail vein injection of 4T1 control cells and mmu-miR-33-expressing cells (top-left). Quantitation of lung metastases as assessed by bioluminescence measurements (top-right) (n = 4). Arrows indicate metastatic foci (bottom). (D) Bioluminescence images of mice showing primary tumors derived from orthotopic injections into the mammary fat pad of MDA-MB-231 control cells (ctrl) and MDA-MB-231/miR-33b cells at day 28 (left). Images of tumors from all mice in each group (middle). Quantitation of tumors from all mice in each group (right) (n = 5). (E) Bioluminescence images of mice showing lung metastases at day 42 derived from orthotopic injections of MDA-MB-231 control cells and miR-33b-expressing cells. In this case, primary tumors were resected at day 28 (top-left). Quantitation of lung metastases as assessed by bioluminescence measurements at day 42 (top-right) (n = 5). Arrows indicate metastatic foci (bottom). (F) Bioluminescence images of mice showing lung metastases at day 42 derived from the tail vein injection of MDA-MB-231 control cells and miR-33b-expressing cells (top-left). Quantitation of lung metastases as assessed by bioluminescence measurements (top-right) (n = 5). Arrows indicate metastatic foci (bottom). The color scale bar depicts the photon flux (photons per second) emitted from these mice. Scale bars, 100 μm. Data represent the mean ± s.d. *: *P* <0.05, **: *P* <0.01, ***: *P* <0.001.

Because our *in vitro* data revealed that miR-33b expression was highly associated with pro-metastatic traits, we further asked whether miR-33b could suppress tumor metastasis in *vivo*. We used highly metastatic mouse breast cancer cells 4T1 to generate luciferase-labeled 4T1/ctrl and 4T1/mmu-miR-33 cells (Supplementary Fig. 6) and injected them into the orthotopic site or tail vein of mice. Mice injected with 4T1/ctrl cells at the mammary fat pad formed tumors with a larger size and a higher weight than mice injected with 4T1/mmu-miR-33 cells at the orthotopic site (Fig. 7A). Bioluminescence imaging showed that mice bearing the 4T1/ctrl cells formed multiple large metastases, while 4T1/mmu-miR-33 cells displayed relatively weak metastasis both in orthotopic implantation and tail vein injection (Fig. 7B,C). We also injected luciferase-labeled MDA-MB-231/miR-33b cells and the control cells into the orthotopic site or tail vein of nude mice. Bioluminescence imaging showing that mice bearing the MDA-MB-231/ctrl cells displayed significant lung metastases and a larger size and a higher weight of tumors; however, we only detected few metastases in the mice injected with MDA-MB-231/miR-33b cells (Fig. 7D–F). Collectively, these data suggest that miR-33b inhibits tumorigenesis and suppresses breast cancer cell metastasis *in vivo*.

## Discussion

miRNAs can regulate various biological processes in tumorigenesis and metastasis^35^,^36^. In this study, we reported that miR-33b was downregulated in breast tumor samples from patients compared with adjacent normal breast tissues. miR-33b expression was inversely correlated with clinical stages and metastatic status of breast cancer. We also found that miR-33b expression was inversely related with the metastatic potential of breast cancer cell lines. Moreover, miR-33b could inhibit tumor growth and the lung metastasis of breast cancer cells *in vivo*. Interestingly, microarray data in a recent report showed that miR-33b expression is decreased with metastatic stage progression in human breast cancer^37^. Therefore, miR-33b may exert tumor-suppressive functions and impede breast tumor metastasis.

CSCs have been reported to participate in various steps of tumor pathogenesis and have the potential to drive tumorigenesis, metastatic growth and recurrence. Interestingly, a recent report demonstrated that miR-33a, another member of the miR-33 family, promotes the self-renewal of glioma-initiating cells^38^. Our study is the first to identify miR-33b as a suppressor of CSC stemness. Using *in silico* analysis and dual-luciferase reporter assays, we identified HMGA2, SALL4 and Twist1 as direct downstream target genes of miR-33b. As a member of high mobility group A proteins, HMGA2 is often overexpressed in various types of human cancers^39^. HMGA2 promotes neural stem cell self-renewal in young mice^40^ and serves as a specific downstream target of Lin28b to regulate mouse hematopoietic stem cell self-renewal^41^. HMGA2 knockdown suppresses breast CSC self-renewal by maintaining the undifferentiated subpopulation^42^. SALL4 is a newly discovered stemness-related oncogene. SALL4 has been identified as a stem cell biomarker in hepatocellular carcinoma^43^ and can upregulate the stemness-related genes KRT19, EpCAM and CD44 in EpCAM-positive hepatocellular carcinoma^44^. SALL4 is also overexpressed in gastric cancer and is linked with the EMT as well as stemness^45^. Overexpression of Twist1 in breast cancer cells induces EMT and generates cells with the properties of stem cells^26^. Our data demonstrated that miR-33b dramatically downregulated the expression of HMGA2, SALL4 and Twist1 in breast cancer cells to suppress cell self-renewal. We also found that HMGA2 knockdown decreased Bmi-1, Nanog, Oct4 and Sox2 expression in MDA-MB-231 cells (Supplementary Fig. 7). Because both SALL4 and Twist1 can upregulate polycomb group protein Bmi-1 at the transcriptional level, miR-33b may simultaneously inhibit the downstream targets SALL4 and Twist1 to further suppress Bmi-1. In addition, miRNA can act pleiotropically; therefore, HMGA2, SALL4 and Twist1 are the ideal targets of miR-33b in modulating the stemness of breast cancer cells. However, miR-33 can downregulate p53 by directly binding to the p53 3′UTR to promote self-renewal in murine hematopoietic stem cells^46^. SALL4 has been reported to regulate Bmi-1 transcription to modulate the self-renewal of normal hematopoietic and leukemic stem cells^47^. Therefore, the discrepancy that miR-33 plays opposing roles in regulating the stemness of hematopoietic stem cells and breast cancer cells remains to be investigated.

In addition to inhibiting the stemness of breast cancer cells, our data demonstrated that miR-33b inhibits metastasis, at least partly, by remodeling the extracellular matrix. miR-33b can decrease the levels of MMP-2, MMP-9, LOX, CXCR4, FN and p-FAK in breast cancer cells by regulating downstream targets. MMP-2, MMP-9, LOX, CXCR4, FN and p-FAK are involved in remodeling the tumor metastatic microenvironment, and whether miR-33b can regulate the metastatic microenvironment deserves further investigation. In addition, although the three target genes of miR-33b, HMGA2, SALL4 and Twist1, are EMT inducers, our gain-of-function and loss-of-function assays in different breast cancer cell lines and a mammary epithelial cell line showed that miR-33b regulation of tumor metastasis is independent of the EMT-related program. Moreover, we found that miR-33b does not alter the 3′UTR activity of the well-established EMT transcription factor SNAI2, suggesting that miR-33b regulates HMGA2, SALL4 and Twist1 in breast cancer cells without impacting EMT.

In summary, our study has demonstrated that miR-33b expression is downregulated in breast tumor samples and is inversely correlated with lymph node metastatic status. miR-33b can inhibit the self-renewal of breast cancer cells and their migration and invasion capabilities by targeting HMGA2, SALL4 and Twist1. Furthermore, miR-33b suppresses the lung metastasis of breast cancer cells *in vivo*. Our results revealed a new miRNA that regulates both stemness and metastasis in breast cancer cells, indicating that miR-33b may serve as a new diagnostic and prognostic biomarker for breast cancer metastasis.

## Methods

### Cell lines and cell culture

The noncancerous human mammary epithelial cell line MCF-10A, breast cancer cell line MCF-7 and human embryonic kidney epithelial cell line 293T were provided by Dr Kunxin Luo (University of California at Berkeley, Berkeley, CA). Human breast cancer cell line MDA-MB-231 was obtained from Dr Guohong Hu (Institute of Health Sciences, Shanghai Institute for Biological Sciences, Shanghai). BT-549, MDA-MB-453, SK-BR-3 and 4T1 cells were obtained from the Institute of Biochemistry and Cell Biology, Chinese Academy of Sciences, Shanghai. MCF-10A cells were maintained as described^48^. 293T and MDA-MB-453 cells were cultured in DMEM supplemented with 10% fetal bovine serum. MCF-7, 4T1, MDA-MB-231, SK-BR-3 and BT-549 cells were maintained in RPMI1640 media supplemented with 10% fetal bovine serum.

### Plasmid construction and generation of stable cell lines

Hsa-miR-33b containing flank region was amplified from human genomic DNA and inserted into pCDH-CMV-EF1α-GFP+puro (System Biosciences). The entire length of the 3'UTR of HMGA2, SALL4, Twist1, ADAM9, SNAI2 or LDHA was cloned into pMIR-REPORT miRNA Expression Reporter Vector (Ambion). For the generation of miR-33b stable cell lines, a lentivirus-mediated packaging system containing four plasmids, pCDH-miR33b or control plasmid (scrambled miRNA), pMDL, REV and VSVG at the ratio (quantity) of 5:5:2:3, was used. For the stable knockdown miR-33b in MCF-10A cells, pLL3.7-puro containing anti-miR-33b, anti-pre-miR-33b shRNA or control plasmid (control hairpin) was co-transfected with pMDL, REV and VSVG at the ratio (quantity) of 5:5:2:3. The transfection and lentiviral infection processes were similar to those described previously^48^. The primers used to generate these constructs are listed in Supplementary information Table S1 and S2.

### Real-time RT–PCR

Total RNA was prepared with the Trizol reagent (Invitrogen) according to the manufacturer’s instructions. For miRNA reverse transcription, cDNA was synthesized using TaqMan® MicroRNA Reverse Transcription Kit (ABI) with 100 ng total RNA. For mRNA reverse transcription, cDNA was synthesized using ReverTra Ace® qPCR RT Kit (TOYOBO) with 1 μg total RNA. Real-time PCRs were performed using SYBR® Select Master Mix for CFX (Invitrogen). Relative quantification was achieved by normalization to the amount of GAPDH or U6. The primers used are shown in Supplementary information Table S3.

### Mammosphere culture

Mammosphere culture was performed as described^48^. Primary, secondary and tertiary mammospheres were seeded at 4,000 cells per well.

### Western blotting

Western blotting was performed as described previously^49^. Cell lysates (10 μg) were subjected to SDS–polyacrylamide gel and immunoblot analysis with antibodies against the following proteins: HMGA2, Sox2 and Vimentin (R&D); SALL4 and Oct4 (Protein Tech.); Nanog, Bmi-1 and β-actin (Millipore); Twsit1, LOX and Fibronectin (Abcam); p-FAK (Invitrogen); E-Cadherin, N-Cadherin and β-catenin (BD); α-E-catenin (Cell Signaling); a-SMA (Sigma Aldrich).

### Migration and invasion assays

Migration and invasion assays were performed as described previously^49^. For the Transwell migration assay, 1 × 10^4 ^MDA-MB-231, 1 × 10^4^ BT-549 or 3 × 10^4 ^MCF-10A cells were plated on the top chambers of 8 μm pore size Transwell plates (Corning). For the Matrigel-coated Transwell invasion assay, Matrigel and 3 × 10^4 ^MDA-MB-231, 3 × 10^4 ^BT-549 or 8 × 10^4 ^MCF-10A cells were plated on the top chambers of 8 μm pore size Transwell plates (Corning). All experiments were performed at least three times in triplicate.

### Flow cytometry

The identification of CD44^high^/CD24^low^ cells in MCF-10A cells was performed using monoclonal anti-CD44-FITC (clone G44-26) and anti-CD24-PE (clone ML5) antibodies (BD Biosciences). For MDA-MB-231 cells, we used anti-CD44-APC (clone IM7) and anti-CD24-APC-eFluor®780 (clone SN3 A5-2H10) antibodies (eBiosciences). The cells were labeled and CD44 and CD24 markers were analyzed using a Fortessa flow cytometer (BD Biosciences).

### miRNA reporter luciferase assay

293T or MDA-MB-231 cells were seeded into a 24-well plate and cotransfected with miR-33b or control and 3′UTR-luciferase plasmids. The cells were lysed at 48 h posttransfection, and the luciferase activity was measured using the Dual-Glo Luciferase Assay System (Promega) and normalized to Renilla luciferase activity.

### Animal studies

This study and all experimental protocols were approved and the methods were carried out in accordance with the guidelines of the Animal Care and Use Committee of Xiamen University. For orthotopic metastasis assays, 1 × 10^6 ^4T1 cells or 3 × 10^6^ MDA-MB-231 cells were injected into the mammary fat pad of six-week-old female BALB/C mice or nude mice (n = 4-5 per group). Following tumor growth detection and resection of primary tumors on day 21 (4T1) or 28 (MDA-MB-231), pulmonary metastasis on day 35 (4T1) or 42 (MDA-MB-231) was monitored by the live animal Lumina II system (Xenogen IVIS system) once per week. For tail vein metastasis, 5× 10^5^ 4T1 cells or 1 × 10^6^ MDA-MB-231 cells were injected into the tail veins of six-week-old female BALB/C mice or nude mice (n = 4-5 per group). Pulmonary metastasis on day 14 (4T1) or 42 (MDA-MB-231) was monitored by the live animal Lumina II system (Xenogen IVIS system) once per week.

### Human samples

For the human tissue samples, the methods were carried out in accordance with the approved guidelines by the Ethics and Scientific Committees of Xiamen University. Human breast tumor tissues and matched normal tissue samples (n = 29) were collected from patients at the Department of Surgical Oncology, First Affiliated Hospital of Xiamen University. All of the samples and matched clinical information were collected after obtaining prior written informed consent from the patients. For the use of clinical materials for research purposes, prior approval was obtained from the Department of Surgical Oncology, First Affiliated Hospital of Xiamen University.

### Statistical analysis

All quantitative data were expressed as the mean ± standard deviation (mean ± s.d.) from at least three samples or experiments per data point. Significant differences were analyzed by Student’s *t*-test to compare two groups of independent samples.

## Author Contributions

**Authors contributions** Y.L., A.Y.L., C.F., H.Z., Y.L., S.W. and D.Y. and performed the experiments. Y.L., A.Y.L. and H.Z. performed bioinformatics analyses. C.Z., Z.H. and Q.L. prepared tumor specimen and mRNA. F.L. and C.J.Y. provided technical support. A.Y.L., Y.L. and G.O. analysed the data and wrote the paper.

## Supplementary Material

Supplementary Information Supplementary information
