# Author Correction: MicroRNA-33b inhibits breast cancer metastasis by targeting HMGA2, SALL4 and Twist1

**DOI:** 10.1038/s41598-025-14334-z

**Published:** 2025-09-01

**Authors:** Yancheng Lin, Allan Yi Liu, Chuannan Fan, Hong Zheng, Yuan Li, Chuankai Zhang, Shasha Wu, Donghong Yu, Zhengjie Huang, Fan Liu, Qi Luo, Chaoyong James Yang, Gaoliang Ouyang

**Affiliations:** 1 State Key Laboratory of Cellular Stress Biology, Innovation Center for Cell Signaling Network, School of Life Sciences, Xiamen University, Xiamen, 361102 China; 2 Department of Surgical Oncology, First Affiliated Hospital of Xiamen University, Xiamen, 361003 China; 3 Medical College, Xiamen University, Xiamen, 361102 China; 4 College of Chemistry and Chemical Engineering, Xiamen University, Xiamen, 361005 China

Correction to: *Scientific Reports* 10.1038/srep09995, published online 28 April 2015

The original Article contains errors. Due to an error during figure assembly, the left two panels in Figure 6G are partially overlapping with the images in Figure 4B. In addition, all images in Figure 6G were selected from cell migration assays instead of representing cell invasion.

The images in Figure 6G have been replaced and the corrected Figure is shown below as Figure 1.

**Fig. 1:**
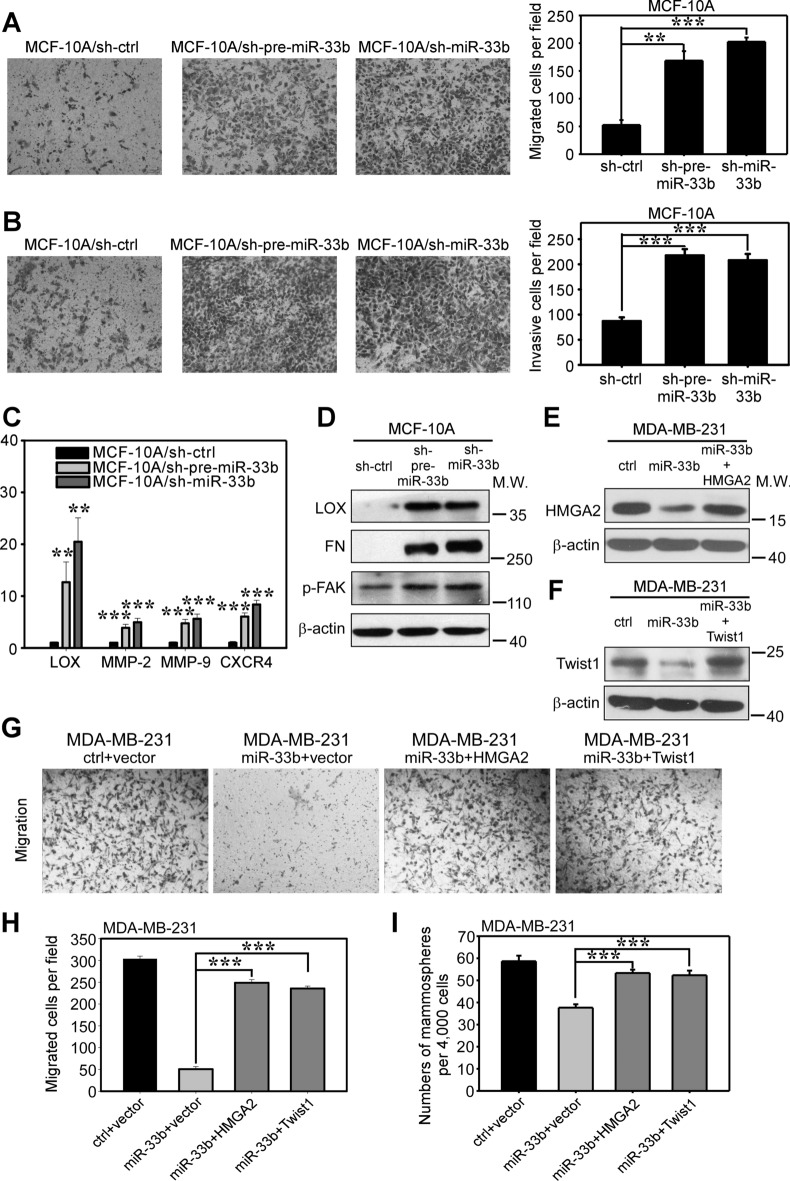
(**A**,**B**) Transwell migration (**A**) and Matrigel-coated Transwell invasion (**B**) analyses revealed that knockdown of miR-33b promoted the migration and invasion of MCF-10A cells *in vitro*. (**C**) qRT-PCR analysis revealed that the knockdown of miR-33b upregulated the mRNA expression of the metastasis-related genes LOX, MMP-2, MMP-9 and CXCR4 in MCF-10A cells. (**D**) Western blot analysis of metastasis-related proteins LOX, FN and p-FAK. These proteins were upregulated after miR-33b knockdown in MCF-10A cells. (**E**,**F**) Western blot analysis of the re-expression of HMGA2 and Twist1 in MDA-MB-231/miR-33b cells. The full-length blots were presented in the Supplementary Figure 11. (**G**) Migration assays with the indicated MDA-MB-231 cells transfected with miRNA-resistant expression constructs. Control represents the scrambled miRNA used for miR-33b overexpression and vector represents the empty vector used for HMGA2 and Twist1 re-expression. (**H**) Quantification of migration assays with the indicated MDA-MB-231 cells transfected with miRNA-resistant expression constructs in (**G**). (**I**) Quantification of mammosphere formation assays with the indicated MDA-MB-231 cells transfected with miRNA-resistant expression constructs. Scale bars, 100 μm. Data represent mean ± s.d. **: *P* < 0.01, ***: *P* < 0.001.

In the Methods section under the subheading ‘Animal studies’ the sentence:

For tail vein metastasis, 5 × 10^5^ 4T1 cells or 1 × 10^6^ MDA-MB-231 cells were injected into the tail veins of six-week-old female BALB/C mice or nude mice (n = 4–5 per group).

Now reads:

For tail vein metastasis, 5 × 10^5^ 4T1 cells or 1 × 10^6^ MDA-MB-231 cells were injected into the tail veins of six-week-old female BALB/C mice or male and female nude mice (n = 4–5 per group).

