# Supplementary material for: MicroRNA-33b Inhibits Breast Cancer Metastasis by Targeting HMGA2, SALL4 and Twist1
Source: Sci Rep. 2015 Apr 28;5:9995. doi: 10.1038/srep09995 (PMC4412117; doi:10.1038/srep09995)
Supplement: Supplementary Information [file srep09995-s1.pdf]

SUPPLEMENTAL INFORMATION

Supplementary Figure 1-11

Supplementary Materials and Methods

Supplementary Table 1-3

MicroRNA-33b Inhibits Breast Cancer Metastasis by Targeting HMGA2, SALL4 and Twist1

Yancheng Lin[1,*], Allan Yi Liu[1,*], Chuannan Fan[1,*], Hong Zheng[1], Yuan Li[1], Chuankai Zhang[1,2], Shasha Wu[1], Donghong Yu[1], Zhengjie Huang[2], Fan Liu[3], Qi Luo[2], Chaoyong James Yang[4] & Gaoliang Ouyang[1]

[1]State Key Laboratory of Cellular Stress Biology, Innovation Center for Cell Signaling Network, School of Life Sciences, Xiamen University, Xiamen 361102, China. [2]Department of Surgical Oncology, First Affiliated Hospital of Xiamen University, Xiamen 361003, China. [3]Medical College, Xiamen University, Xiamen 361102, China. [4]College of Chemistry and Chemical Engineering, Xiamen University, Xiamen 361005, China.

*These authors contributed equally to this work.

Correspondence and requests for materials should be addressed to G.O. (email: oygldz@xmu.edu.cn)

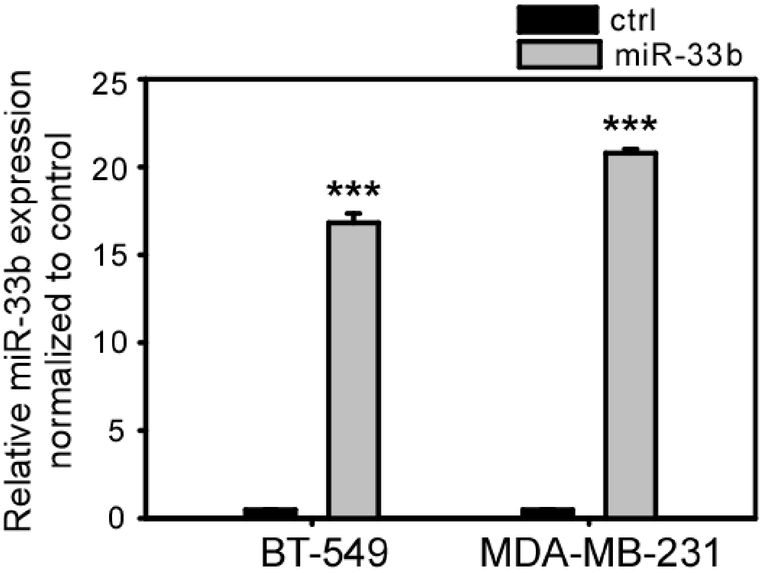

Supplementary Figure 1. (A) qRT-PCR analysis of the miR-33b expression levels after the ectopic expression of miR-33b in BT-549 and MDA-MB-231 cells. Data represent the mean ± s.d. ***: P <0.001.

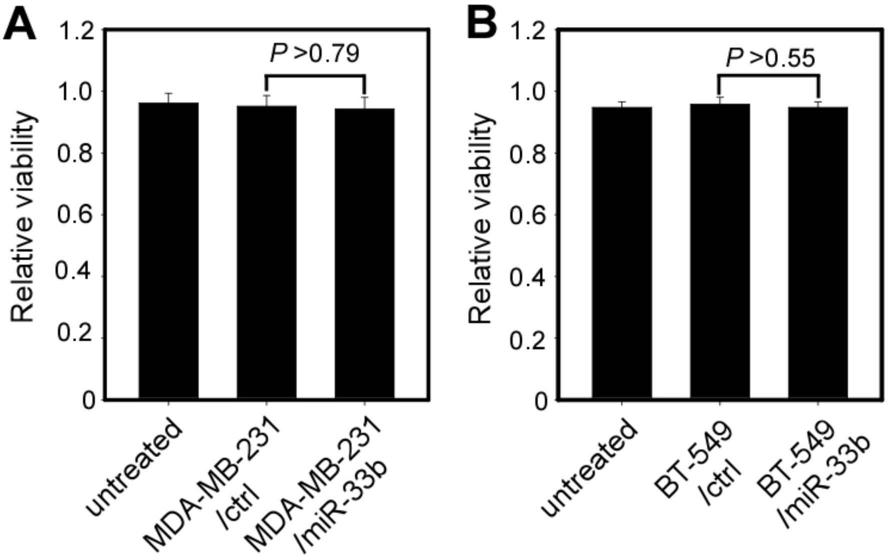

Supplementary Figure 2. Ectopic expression does not cause toxicity in breast cancer cells. Trypan blue dye exclusion assay was used to determine the effect of ectopic expression of miR-33b on the viability of BT-549 (A) or MDA-MB-231 (B) for 48 h.

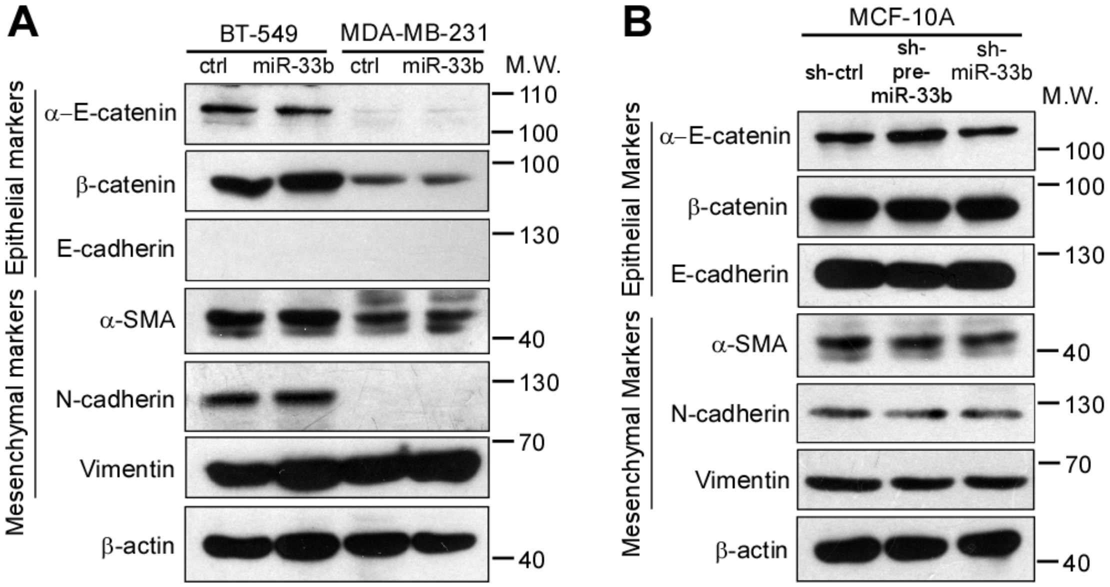

Supplementary Figure 3. The effects of ectopic miR-33b on the expression of EMT markers in BT-549, MDA-MB-231 (A) and MCF-10A (B) cells were determined by western blotting.

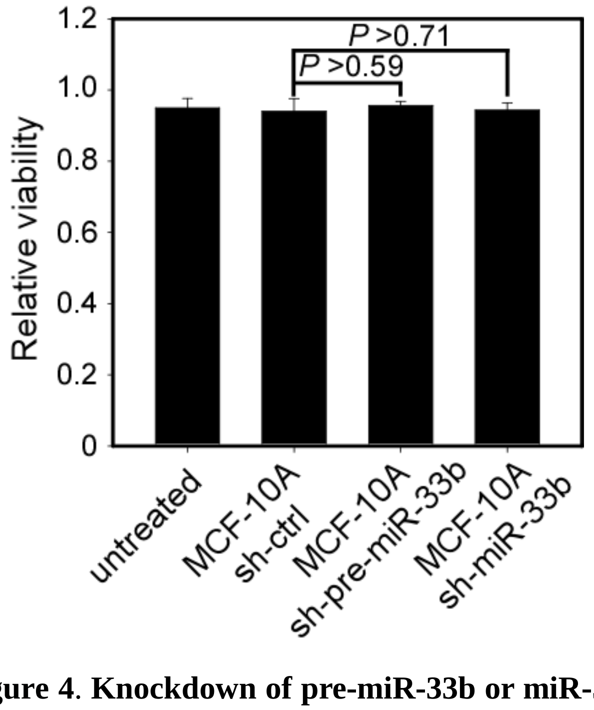

Supplementary Figure 4. **Knockdown of pre-miR-33b or miR-33b does not cause toxicity on MCF-10A cells.** Trypan blue dye exclusion assay was used to determine the effect of the knockdown of miR-33b on the viability of MCF-10A cells for 48 h.

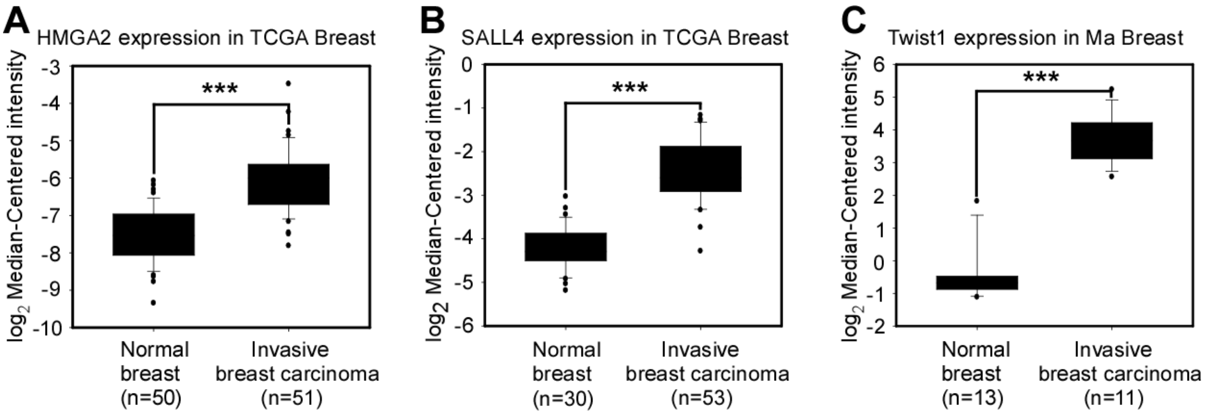

Supplementary Figure 5. Oncomine data analyses of the expression of HMGA2, SALL4 and Twist1 mRNAs in invasive breast carcinoma tissues and normal breast tissues. (A) Expression of HMGA2 mRNA levels in invasive breast carcinoma (n=51) compared with normal breast tissue (n = 50). Data were obtained from the Cancer Genome Atlas (TCGA) breast cancer gene-expression data set. (B) Expression of SALL4 mRNA levels in invasive breast carcinoma (n = 53) compared with normal breast tissue (n=30). Data were obtained from the TCGA breast cancer gene-expression data set. The Y axis in the graph uses a log2 scale. (C) Expression of Twist1 mRNA levels in invasive breast carcinoma (n = 11) compared with normal breast tissue (n = 13). Data were obtained from Ma breast (GEO/GSM3639). Data represent the mean ± s.d. ***: *P* <0.001.

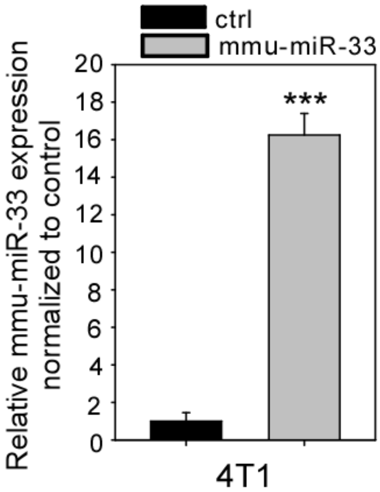

Supplementary Figure 6. qRT-PCR analysis of the mmu-miR-33 expression levels after the ectopic expression of mmu-miR-33 in mouse 4T1 cells. Data represent the mean ± s.d. ***: *P* <0.001.

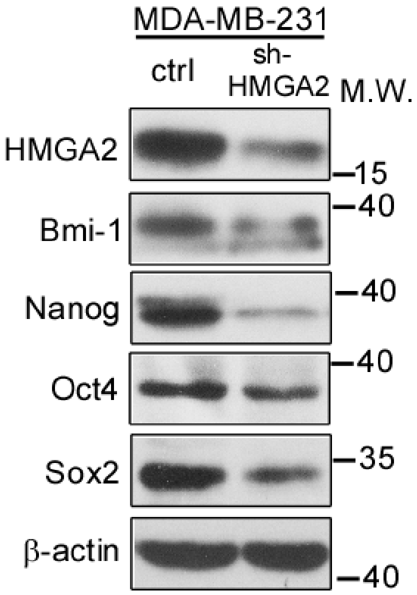

Supplementary Figure 7. Western blot analysis of the expression of HMGA2 and stemness-related proteins Bmi-1, Nanog, Oct4 and Sox2 upon knockdown of HMGA2 in MDA-MB-231 cells.

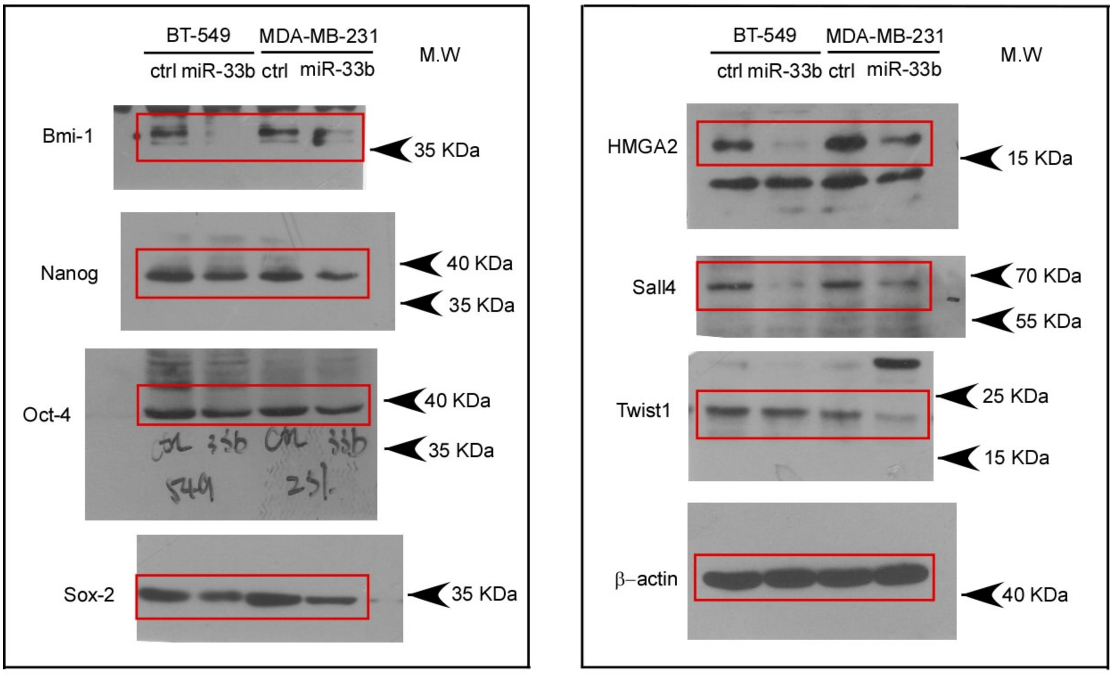

Figure 3G

Supplementary Figure 8. Full-length images of the immunoblots. Red line boxes indicate the cropped images used in Figure 3G. β-actin was used as an internal control. The same set of protein samples was charged in all gels. Arrowheads indicate the position of protein markers.

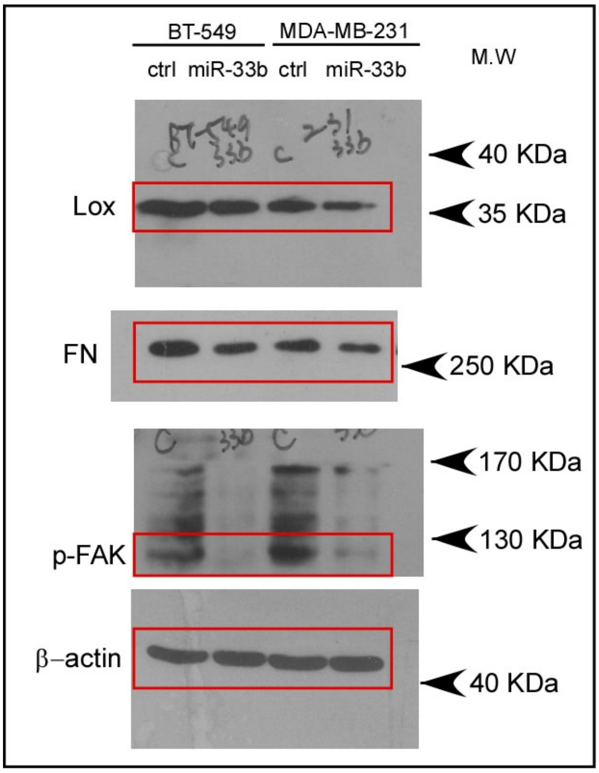

Figure 4F

Supplementary Figure 9. Full-length images of the immunoblots. Red line boxes indicate the cropped images used in Figure 4F. β-actin was used as an internal control. The same set of protein samples was charged in all gels. Arrowheads indicate the position of protein markers.

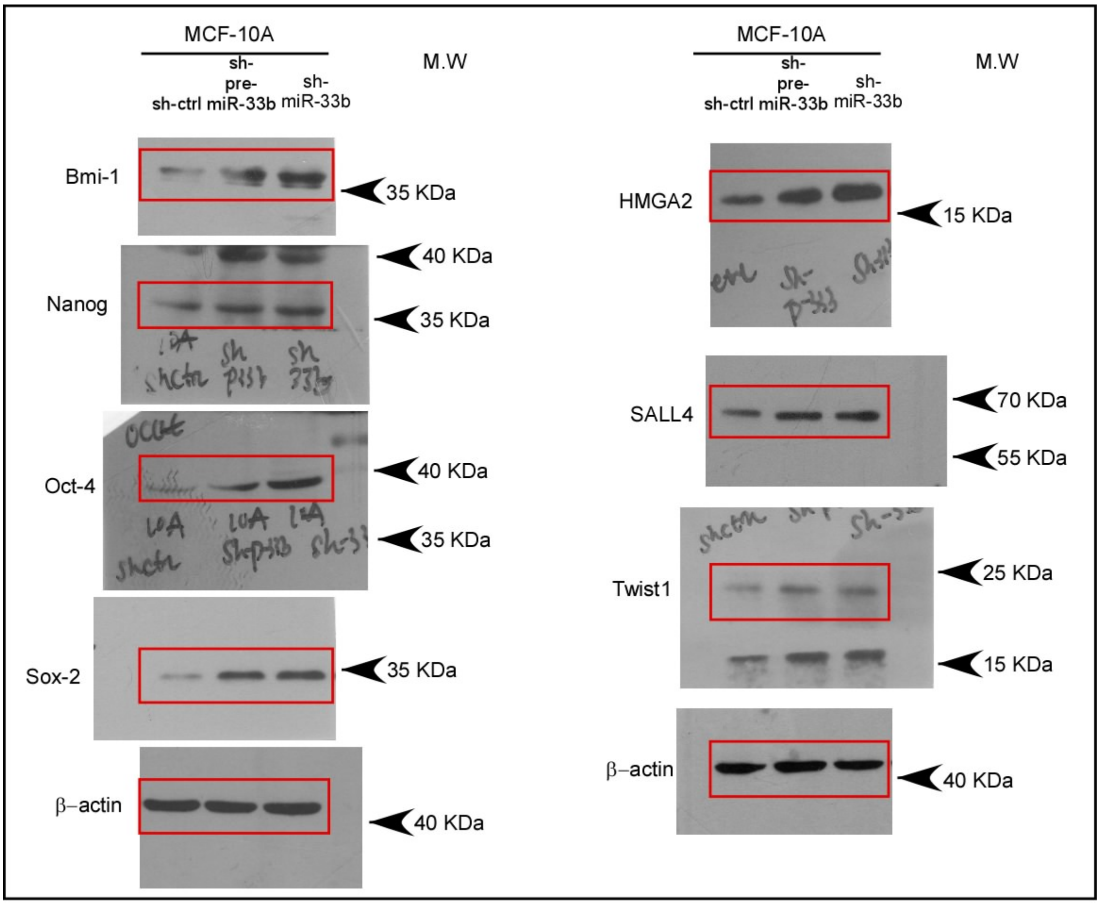

Figure 5G

Supplementary Figure 10. Full-length images of the immunoblots. Red line boxes indicate the cropped images used in Figure 5G. β-actin was used as an internal control. The same set of protein samples was charged in all gels. Arrowheads indicate the position of protein markers.

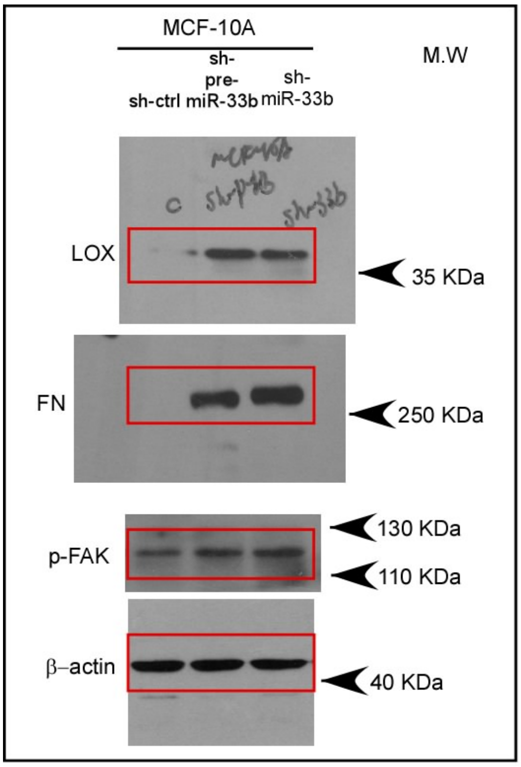

Figure 6D

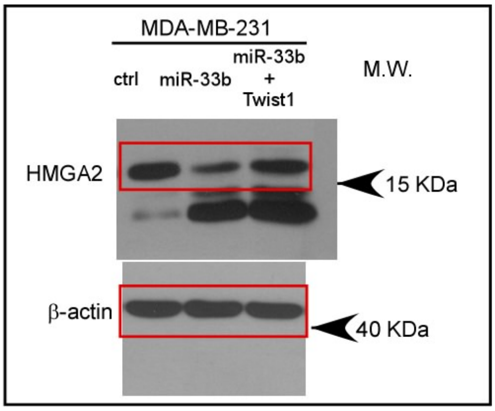

Figure 6E

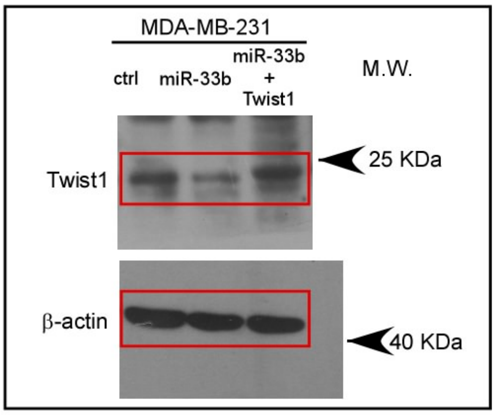

Figure 6F

Supplementary Figure 11. Full-length images of the immunoblots. Red line boxes indicate the cropped images used in Figure 6D, E and F. β-actin was used as an internal control. The same set of protein samples was charged in all gels. Arrowheads indicate the position of protein markers.

Supplementary Materials and Methods

Plasmid construction. To generate the miR-33b overexpression construct, a 415-bp genomic fragment covering the region encoding primiR-33b and its up and downstream regions were PCR amplified and inserted into pCDH-CMV-EF1-GFP+puro (System Biosciences). The full-length sequence was as follows:

AGCCCAAGCAGCACAGGATAGGGGGTCCAGCAGACCCTGCTTTTTGGCTA

AGGCTTCTGTCCAGAGGAGAGGGGTTGCCCCTATCTGGCCTCAGTTTCCCC

ATCCCTGGGAGGAGGGGGGTGGATGGTGTGGTAGGATCCCTTTGGAGGCC CTGCATCAGGAGGGCTGGACAGCTGCTCCCGGGCCGGTGGCGGGTGTGGG GGCCGAGAGAGGCGGGCGGCCCCGCGGTGCATTGCTGTTGCATTGCACGT GTGTGAGGCGGGTGCAGTGCCTCGGCAGTGCAGCCCGGAGCCGGCCCCTG GCACCACGGGCCCCCATCCTGCCCCTCCCAGAGCTGGAGCCCTGGTGACC CCTGCCCTGCCTGCCACCCCCAGGCCGTGCAGCTGTTCCTGTGTGACCTGC TTCTTGTGGTGCG. To generate the miRNA-insensitive HMGA2 and Twist1 constructs, the region of the human HMGA2 CDS (NM_003483.4) at 812–1141 bp and the Twist1 CDS (NM_000474.3) at 352–960 bp were generated by PCR amplification and subcloned into the pCMV-5 plasmid vector (Addgene). For the stable knockdown of miR-33b in MCF-10A cells, the best hairpin sequences targeting miR-33b and pre-miR-33b used were GCATTGCTGTTGCATTGC (anti-miR-33b) and TGAGGCGGGTGCAGTGCC (anti-pre-miR-33b).

Cell viability assays. The impact of transient transfection with various constructs and reagents on cell viability was assessed by the Trypan blue dye exclusion assay. Briefly, 2.0×10^5 cells were seeded in 6-well plates, and then were transfected with the indicated expression constructs. After 48 h, cells were trypsinized, resuspended in 0.4% Trypan blue staining solution (Sigma) and manually counted using a hemocytometer. The viability was expressed as the percentage of the viable cell number.

Oncomine analysis. The Oncomine database was used to analyze the HMGA2, SALL4 and Twist1 genes. The data sets containing expression data for these three genes were filtered to display downregulation in breast carcinoma versus normal breast tissue with $P < 0.05$.

Table S1 The sequences of plasmid constructs

Has-miR-33b	Fw<br>Rv	AGCCCAAGCAGCACAGGATAGG<br>CGCACCACAAGAAGCAGGTCAC
Mouse-miR-33	Fw<br>Rv	CTCTTACTCTCAGGTCCTGCTAG<br>AGGAAGCAGCTAGTTCTA ACCTC
HMGA2 3′UTR	Fw<br>Rv	GAGTCTGCCGAAGAGGACTAG<br>AATGACTTGTGAGTGTCTCCC
Twist1 3′UTR	Fw<br>Rv	AGCAGGGCCGGAGACCTAGATG<br>AATATAGACCAAACTCTAAG
SALL4 3′UTR	Fw<br>Rv	GGGAGAACTTGCGTGGAAGGAG<br>GCAAAGCAGCATAGCAACAATCG
ADAM9 3′UTR	Fw<br>Rv	TCAGGGAACTGAGCTAATAC<br>CTTGATTGTTAAAATAACATGCTGTC
LDHA 3′UTR	Fw<br>Rv	AGGATGATGTCTTCCTTAGTGTTC<br>CTGCACCCTGCCTTAAGATTG
SNAI2 3′UTR	Fw<br>Rv	GTGACGCAATCAATGTTTACTCG<br>CGTCATGTCTCTTCTGAATGTG

Table S2 The sequences of shRNA constructs

shHMGA2	Fw	TGTCCCTCTAAAGCAGCTCATTCAAGAGATG AGCTGCTTTAGAGGGACTTTTTC
	Rv	TCGAGAAAAAGTCCCTCTAAAGCAGCTCAT CTCTTGAATGAGCTGCTTTAGAGGGACA
sh-miR-33b	Fw	TGCATTGCTGTTGCATTGCTTCAAGAGAGCAA TGCAACAGCAATGCTTTTTC
	Rv	TCGAGAAAAAGCATTGCTGTTGCATTGCTCT CTTGAAGCAATGCAACAGCAATGCA
sh-pre-miR-33b	Fw<br>Rv	TGTGAGGCGGGTGCAGTGCCTTCAAGAGAGG CACTGCACCCGCCTCACTTTTTC
	Fw<br>Rv	TCGAGAAAAAGTGAGGCGGGTGCAGTGCCT CTCTTGAAGGCACTGCACCCGCCTCACA

Table S3 Primer sequences for qRT-PCR analyses

miR-33b reverse	Reverse primer	GTCGTATCCAGTGCGTGTCGTGGAGTCGGCAA T TGCACTGGATACGACTGCAATG
miR-33b Real-time	Fw<br>Rv	GGGGGTGCATTGCTGTTG<br>TGCGTGTCGTGGAGTC

U6-reverse	Reverse primer	CGCTTCACGAATTTGCGTGTCAT
U6-real-time	Fw	GCTTCGGCAGCACATATACTAAAAT
	Rv	CGCTTCACGAATTTGCGTGTCAT
SOX2	Fw	TACAGCATGTCCTACTCGCAG
	Rv	GAGGAAGAGGTAACCACAGGG
MMP2	Fw	GCCCCAGACAGGTGATCTTG
	Rv	GCTTGCGAGGGAAGAAGTTGT
MMP9	Fw	AGACGGGTATCCCTTCGACG
	Rv	AAACCGAGTTGGAACCACGAC
OCT4	Fw	CTTGCTGCAGAAGTGGGTGGAGGAA
	Rv	CTGCAGTGTGGGTTTCGGGCA
Nanog	Fw	AATACCTCAGCCTCCAGCAGATG
	Rv	TGCGTCACACCATTGCTATTCTTC
Bmi-1	Fw	TGGAGAAGGAATGGTCCACTTC
	Rv	GTGAGGAAACTGTGGATGAGGA
LOX	Fw	GCATACAGGGCAGATGTCAGA
	Rv	TTGGCATCAAGCAGGTCATAG
SALL4	Fw	GAGTATCAGAGCCGAAGCCC
	Rv	AGACTGCTCCGACCTTCCAT
HMGA2	Fw	CAAGTTGTTCAGAAGAAGCCTGC
	Rv	CATGGCAATA CAGAATAAGT GGTCAC
GADPH	Fw	GGTCTCCTCTGACTTCAACA
	Rv	GTGAGGGTCTCTCTCTTCCT
Twist1	Fw	GTCCGCAGTCTTACGAGGAG
	Rv	GCTTGAGGGTCTGAATCTTGCT
CXCR4	Fw	CAGCAGGTAGCAAAGTGACG
	Rv	CAGGGTTCCTTCATGGAGTC
RAC1	Fw	CCTGATGCAGGCCATCAAG
	Rv	AGTAGGGATATATTCTCCAGGAAATGC
YES1	Fw	CCAGGTATGGTGAACCGTGAA
	Rv	CCTGTATCCTCGCTCCACTTGT
ZEB1	Fw	AACCCAACTTGAACGTCACA
	Rv	ATTACACCCAGACTGCGTCA
ADAM9	Fw	GGTGACAGATTTGGCAATTGTG
	Rv	TTGTGCCTTCGTTAACCATCC
HIF1α	Fw	CATAAAGTCTGCAACATGGAAGGT
	Rv	ATTTGATGGGTGAGGAATGGGTT
LDHA	Fw	ACCCAGTTTCCACCATGATT
	Rv	CCCAAAATGCAAGGAACACT
SNAI2	Fw	CCAAACTACAGCGAACTGGA
	Rv	GTGGTATGACAGGCATGGAG
